# The Effects of Family Socioeconomic Status on Psychological and Neural Mechanisms as Well as Their Sex Differences

**DOI:** 10.3389/fnhum.2018.00543

**Published:** 2019-01-18

**Authors:** Hikaru Takeuchi, Yasuyuki Taki, Rui Nouchi, Ryoishi Yokoyama, Yuka Kotozaki, Seishu Nakagawa, Atsushi Sekiguchi, Kunio Iizuka, Yuki Yamamoto, Sugiko Hanawa, Tsuyoshi Araki, Carlos Makoto Miyauchi, Kohei Sakaki, Takayuki Nozawa, Shigeyuki Ikeda, Susumu Yokota, Daniele Magistro, Yuko Sassa, Ryuta Kawashima

**Affiliations:** ^1^Division of Developmental Cognitive Neuroscience, Institute of Development, Aging and Cancer, Tohoku University, Sendai, Japan; ^2^Division of Medical Neuroimaging Analysis, Department of Community Medical Supports, Tohoku Medical Megabank Organization, Tohoku University, Sendai, Japan; ^3^Department of Radiology and Nuclear Medicine, Institute of Development, Aging and Cancer, Tohoku University, Sendai, Japan; ^4^Creative Interdisciplinary Research Division, Frontier Research Institute for Interdisciplinary Sciences, Tohoku University, Sendai, Japan; ^5^Human and Social Response Research Division, International Research Institute of Disaster Science, Tohoku University, Sendai, Japan; ^6^Smart Ageing International Research Center, Institute of Development, Aging and Cancer, Tohoku University, Sendai, Japan; ^7^School of Medicine, Kobe University, Kobe, Japan; ^8^Division of Clinical Research, Medical-Industrial Translational Research Center, School of Medicine, Fukushima Medical University, Fukushima, Japan; ^9^Department of Functional Brain Science, Institute of Development, Aging and Cancer, Tohoku University, Sendai, Japan; ^10^Division of Psychiatry, Tohoku Medical and Pharmaceutical University, Sendai, Japan; ^11^Department of Behavioral Medicine, National Institute of Mental Health, National Center of Neurology and Psychiatry, Tokyo, Japan; ^12^Department of Psychiatry, Tohoku University School of Medicine, Sendai, Japan; ^13^Advantage Risk Management Co., Ltd., Tokyo, Japan; ^14^Department of General Systems Studies, Graduate School of Arts and Sciences, The University of Tokyo, Tokyo, Japan; ^15^Department of Ubiquitous Sensing, Institute of Development, Aging and Cancer, Tohoku University, Sendai, Japan; ^16^Department of Sport Science, School of Science and Technology, Nottingham Trent University, Nottingham, United Kingdom

**Keywords:** family social economic status, voxel-based morphometry, diffusion tensor imaging, sex difference, family income, parents’ highest educational qualification

## Abstract

Family socioeconomic status (SES) is an important factor that affects an individual’s neural and cognitive development. The two novel aims of this study were to reveal (a) the effects of family SES on mean diffusivity (MD) using diffusion tensor imaging given the characteristic property of MD to reflect neural plasticity and development and (b) the sex differences in SES effects. In a study cohort of 1,216 normal young adults, we failed to find significant main effects of family SES on MD; however, previously observed main effects of family SES on regional gray matter volume and fractional anisotropy (FA) were partly replicated. We found a significant effect of the interaction between sex and family income on MD in the thalamus as well as significant effects of the interaction between sex and parents’ educational qualification (year’s of education) on MD and FA in the body of the corpus callosum as well as white matter areas between the anterior cingulate cortex and lateral prefrontal cortex. These results suggest the sex-specific associations of family SES with neural and/or cognitive mechanisms particularly in neural tissues in brain areas that play key roles in basic information processing and higher-order cognitive processes in a way females with greater family SES level show imaging outcome measures that have been associated with more neural tissues (such as greater FA and lower MD) and males showed opposite.

## Introduction

Family socioeconomic status (SES, particularly, family income and parents’ educational qualifications) is an important factor that affects an individual’s neural and cognitive development (Hair et al., 2015; Noble et al., 2015). For example, in young individuals, higher family SES is associated with better cognitive and memory functions, including better working memory, executive function, language and literacy abilities, and memory functions (Bowey, 1995; Noble et al., 2007). Higher family SES is also associated with increased self-regulatory behaviors, academic performance, and sense of well-being and less impulsive decision making, learned helplessness, stress, and psychological distress in young individuals (Evans and English, 2002; Evans et al., 2005; Sirin, 2005; Sweitzer et al., 2008).

Although many previous studies have focused on the effects of SES on these cognitive measures across sexes (e.g., Evans and English, 2002; Evans et al., 2005; Sweitzer et al., 2008), some studies have focused on the sex differences of effects of SES on health measures as described below. It is revealed that higher educational qualification of parents leads to low blood pressure during the developmental phase only in women (Janicki-Deverts et al., 2012). Several studies have shown that the associations between higher childhood SES and lower cardiovascular disease morbidity and mortality in later life are stronger in women than in men (Claussen et al., 2003; Hamil-Luker and Angela, 2007; Næss et al., 2007). In addition, while the effects of family SES on cognitive mechanisms are largely assumed to be mediated by the associations between increased stress and low family SES (Evans and English, 2002; Evans and Schamberg, 2009; Ursache and Noble, 2016a), previous animal studies have shown that females and males show opposite neural changes in response to stressors (Shors et al., 2001). For example, Shors et al. (2001) showed in response to stress event, spine density was enhanced in the male hippocampus but reduced in the female hippocampus. Notably, women tend to have more stressors, particularly socially, and these stressors tend to cause more depressive symptoms (Hankin et al., 2007).

Previous neuroimaging studies have investigated brain functions and brain volume correlates of family SES in young subjects of both sexes. Functional imaging studies have investigated the associations between family SES and a wide range of cognitive tasks. Based on these studies, SES is suggested to effect areas of cognitive control and regulation (prefrontal cortex), social emotional processing (amygdala), and memory (hippocampus) as well as the left hemisphere language network for language-related processing (Noble et al., 2006; Gianaros et al., 2008; Kishiyama et al., 2009; Hanson et al., 2011; Ursache and Noble, 2016a).

A neuroimaging study with 1099 typically developing individuals aged between 3 and 20 years showed that parents’ educational qualifications and the family income were positively correlated with the total brain surface area (Hurt and Betancourt, 2015). Another study involving 389 typically developing children showed an association between a higher family SES and a greater total gray matter volume (Hair et al., 2015). Although studies with smaller sample sizes have generated inconsistent results, they generally show a relation between low family SES and decreased brain volume (Hanson et al., 2011; Lawson et al., 2013; Luby et al., 2013). Another study involving a diverse sample of 1082 children and adolescents aged between 3 and 21 years showed that a higher family income is related to higher fractional anisotropy (FA) (which reflects the structural properties of white matter) in and near the hippocampus and frontal cortex (Ursache and Noble, 2016b).

Mean diffusivity (MD) is measured by diffusion tensor imaging (DTI) (Beaulieu, 2002) and used to measure the microstructural properties of gray and white matter. As we summarized previously (Takeuchi et al., 2016a), lower MD is sensitive to greater tissue density of the brain parenchyma (though is not strictly a measure of it). Tissue density increases with the increased presence of unspecific cellular structures (i.e., capillaries, synapses, spines, and macromolecular proteins); the properties of myelin, neuronal membranes, and axons; the shape of neurons and glia; and enhanced tissue organization (Beaulieu, 2002; Sagi et al., 2012). The majority of these tissue differences are thought to affect neural plasticity. Therefore, MD is supposed to provide characteristic information regarding neural plasticity (though, it is obviously not limited to be a measure of neural plasticity); indeed, MD measurements served as a characteristic and sensitive tool to study neural plasticity and development (Sagi et al., 2012; Takeuchi et al., 2015a, 2016a).

As described, family SES is an important factor that affects cognitive, socioemotional, neural, and health development. However, despite the characteristic importance of family SES in humans and studies investigating effects of family SES on cognitive and functional and structural neural mechanisms as described above, the following issues have not been investigated: (a) the effects of family SES on MD despite the characteristic property of MD to reflect neural plasticity and development and (b) the influence of sex on the effects of SES, particularly regarding neural mechanisms despite the extensive evidence on sex-specific SES effects on health (low SES levels tended to be associated with more health problems in females than in males as described above).

Therefore, the purpose of this study was to investigate these issues. To this end, we hypothesized that higher family SES is associated with lower MD in the brain areas, including the prefrontal cortex, hippocampus, amygdala, and the left hemisphere language network, and that higher family SES results in more neural tissue, as evidenced by associations with brain volume, FA, and MD more strongly in females.

We also investigated the effects of family SES on relevant psychological measures to reveal the nature of correlates of family SES. Based on abovementioned previous studies and theoretical background of family SES, these include diverse psychological measures of (a) basic cognitive functions, (b) traits related to affects, (c) stress, (d) traits related to cognition, and education. Family SES was separately measured using both family annual income and the parents’ average highest educational qualifications.

## Materials and Methods

### Subjects

The present study, which is a part of an ongoing project to investigate the association between brain imaging, cognitive function, and aging, included relevant SES measures and imaging data from 1216 healthy, right-handed individuals (702 men and 514 women). The mean age of the subjects was 20.7 years [standard deviation (SD), 1.8; age range: 18–27 years old]. For details of subjects’ information, see Supplemental Methods. Written informed consent was obtained. All methods were performed in accordance with the Declaration of Helsinki (1991). This study was approved by the Ethics Committee of Medical Faculty of Tohoku University.

### SES Measures

Data related to the socioeconomic status were collected in accordance with our previous study and mostly with the standard approach used by the Japanese government for evaluating socioeconomic status. Descriptions in this subsection were largely reproduced from our previous study that used similar methods (Takeuchi et al., 2014a). The measure of socioeconomic status consisted of three questions. One was an enquiry relating to family annual income (income of the family that the individual grew up in). Annual income data were collected using discrete variables: 1, annual income <2 million yen (the currency exchange rate is now approximately $1 USD = 120 yen); 2, annual income 2–4 million yen; 3, annual income 4–6 million yen; 4, annual income 6–8 million yen; 5, annual income 8–10 million yen; 6, annual income 10–12 million yen; 7, annual income ≥12 million yen. The values 1–7 were used in subsequent regression analyses. The other two questions related to the highest educational qualification of both parents. There were 8 options [1, elementary school graduate or below; 2, junior high school graduate; 3, graduate of a short term school completed after junior high school; 4, normal high school graduate; 5, graduate of a short term school completed after high school (such as a junior college); 6, university graduate; 7, Masters degree; and 8, Doctorate] and each choice was converted into the number of years taken to complete the qualification in the normal manner in the Japanese education system (1, 6 years; 2, 9 years; 3, 11 years; 4, 12 years; 5, 14 years; 6, 16 years; 7, 18 years; 8, 21 years). The average of the converted values for each parent was used in the analyses. Not all subjects had both parents’ information and in such cases, one parent’s information was used in the analysis. This protocol followed the standard approach used by the Japanese government for evaluating socioeconomic status, but the questions relating to the parents’ highest educational qualifications were modified to increase the number of options available and thus also increased precision. Although, we have no way of knowing whether subjects answered questions sincerely, like in the cases of any other questionnaires, but subjects were explicitly instructed that they are allowed not to answer questions, if they don’t like to. It should also be noted that while parents’ highest educational qualifications may usually not change over time during the development of children, family income may change more or less as have been the cases of most of the studies of family SES.

Instead of composite scores, two SES measures were separately used because most (though not all) relevant previous studies either separated two psychological factors or used one of two measures. Moreover, the effects of two factors are often different in previous studies (Noble et al., 2012; Lawson et al., 2013; Hurt and Betancourt, 2015; Noble et al., 2015).

### Psychological Measures

The following neuropsychological tests and questionnaires were administered to study participants: The references and additional details of these measures were provided in the Supplemental Methods. As described in the Introduction, based on nature of family SES, and previous studies, we investigated the effects of family SES on diverse psychological measures of (a) basic cognitive functions, (b) traits related to affects, (c) stress, (d) traits related to cognition, and education.

(A) Raven’s Advanced Progressive Matrices (RAPM), a non-verbal reasoning task; (B) Tanaka B-type intelligence test (TBIT) type 3B, an intelligence test that does not require verbal knowledge; (C) a reading comprehension task; (D) the S-A creativity test, a measure of creativity measured by divergent thinking (CMDT); (E) computerized digit span task, a verbal (working memory) WM task; (F) SQ and EQ questionnaires, a measure of empathizing (drive to identify the mental status of other individuals) and systemizing (drive to analyze a system); (F) Emotional Intelligence Scale, a questionnaire measure of emotional intelligence; (G) General Health Questionnaire 30 (GHQ30), a measure of mental health; (H) WHOQOL-26, the Japanese version of the QOL Scale; (I) the scale for Critical Thinking Disposition, the measure for evaluating disposition toward critical thinking. (J) the cognitive reflectivity–impulsiveness questionnaire, a measure of individual differences in reflectivity and impulsivity; (K) the Japanese version of Need for Cognition Scale, a measure of the tendency for an individual to engage in and enjoy thinking; (L) the Japanese version of the Achievement Motivation Scale, a measure of two psychometrically derived achievement motivations, including self-fulfillment achievement motivation (SFAM: achievement motivation directed at pursuing goals evaluated by one’s own standards of achievement, regardless of the values of others and the society) and competitive achievement motivation CAM: achievement motivation directed at seeking social prestige by defeating others and achieving better results than others; (M) the Japanese version of the Rosenberg Self-Esteem Scale (RSES), a measure of global trait self-esteem; (N) the National Identity Scale, a self-reported measure of individual nationalism and patriotism; (O) the Japanese version of the Optimism Scale, a questionnaire measure of individual optimism and pessimism; (P) the Japanese version of the General Self-Efficacy Scale, a measure of individual general self-efficacy (general self-efficacy is defined as individuals’ perception of their ability to perform across various different situations); (Q) the Japanese version of the third version of the UCLA Loneliness Scale, a measure of social isolation and loneliness; (R) the Japanese version of the checklist individual strength (CIS) questionnaire, a measure of chronic fatigue; (S) the Japanese version of the Beck Depression Inventory, a measure of states of depression; (T) the Japanese version of State-Trait Anxiety Inventory (STAI), a measure of state and trait anxiety; (U) the shortened Japanese version of the Profile of Mood States (POMS) questionnaire, a measure of participants’ mood in the preceding week [the total score (total mood disturbance) was used in this study]; (V) the Japanese version of the NEO Five-Factor Inventory (NEO-FFI), a measure of five basic personalities: neuroticism, extraversion, openness, agreeableness, and conscientiousness; (W) the Japanese version of Dispositional Envy Scale, a measure of proneness to envy; (X) the Preoccupation Scale, including the External-Preoccupation Scale, which measures the maintenance of external focus on a specific object, and the Self-Preoccupation Scale, which reflects the degree and duration of self-focusing; (Y) the Scale of Egalitarian Sex Role Attitudes-Short Form (SESRA-S), a self-report questionnaire used to measure an individual’s sex-role egalitarianism (SRE), i.e., the belief that the sex of an individual should not influence the perception of his/her rights, abilities, obligations, and opportunities; and (Z) scale of life events in interpersonal and achievement domains, a measure of negative and positive events.

### Behavioral Data Analysis

The behavioral data were analyzed using SPSS 22.0 statistical software (SPSS Inc., Chicago, IL, United States). Some of the descriptions in this subsection were mostly reproduced from our previous study (Takeuchi et al., 2015b).

Associations between SES measures and psychological variables were analyzed using analyses of covariance (ANCOVAs), with age and sex as covariates. To model these analyses, age and one SES measure (family annual income or the parents’ average highest educational qualifications) were used as covariates, sex was a fixed factor, and the interaction between sex and each SES measure was included. Thus, we performed 78 ANCOVAs [number of SES measures (2) × number of psychological measures (39)]. In this study, we investigated the main effects of SES measures as well as the interaction effects of sex and SES measures on diverse neurocognitive mechanisms. Family, annual income and the parents’ average highest educational qualifications were not included in the same model because they are highly correlated and conceptually overlap. This procedure of separate analyses for family income and parents’ education levels has previously performed in this field (Noble et al., 2015).

In all ACNOVAs performed to analyze behavioral measures, results with a threshold of *P* < 0.05 were considered to be statistically significant, after correcting for the false discovery rate (FDR) using the graphically sharpened method (Benjamini and Hochberg, 2000). For the rationale and basic explanation of the FDR test, see Supplemental Methods.

### Image Acquisition

The methods for MR image acquisition were described in our previous studies and reproduced below (Takeuchi et al., 2012; Takeuchi et al., 2016a). All MRI data acquisition was performed using a 3-T Philips Achieva scanner. High-resolution T1-weighted structural images (T1WIs: 240 × 240 matrix, TR = 6.5 ms, TE = 3 ms, FOV = 24 cm, slices = 162, slice thickness = 1.0 mm) were collected using a magnetization-prepared rapid gradient echo sequence from 1216 subjects. Diffusion-weighted data were acquired using a spin-echo EPI sequence (TR = 10293 ms, TE = 55 ms, FOV = 22.4 cm, 2 × 2 × 2 mm^3^ voxels, 60 slices, SENSE reduction factor = 2, number of acquisitions = 1) from 1205 subjects. The diffusion weighting was isotropically distributed along 32 directions (*b*-value = 1,000 s/mm^2^). Additionally, three images with no diffusion weighting (*b*-value = 0 s/mm2) (*b* = 0 images) were acquired, (TR = 10293 ms, TE = 55 ms, FOV = 22.4 cm, 2 × 2 × 2 mm^3^ voxels, 60 slices). FA and MD maps were calculated from the collected images using a commercially available Philips’ diffusion tensor analysis package on the MR console. Images with artifacts were removed after visual inspection and remaining data included images of 1205 subjects as described above. For more details, see Supplemental Methods.

### Pre-processing of Structural Data

Preprocessing of the T1WIs data was performed using Statistical Parametric Mapping software (SPM12; Wellcome Department of Cognitive Neurology, London, United Kingdom) implemented in Matlab (Mathworks Inc., Natick, MA, United States). The methods for the preprocessing of T1WIs were described in our previous studies and reproduced below (Takeuchi et al., 2018). Using the new segmentation algorithm implemented in SPM12, T1-weighted structural images of each individual were segmented and normalized to the Montreal Neurological Institute (MNI) space to give images with 1.5 × 1.5 × 1.5 mm^3^ voxels using diffeomorphic anatomical registration through exponentiated lie algebra (DARTEL) registration process implemented in SPM12. In addition, we performed a volume change correction (modulation) (Ashburner and Friston, 2000). Subsequently, generated rGMV images were smoothed by convolving them with an isotropic Gaussian kernel of 8 mm full width at half maximum (FWHM). For full descriptions of these procedures, see Supplemental Methods.

Preprocessing and analysis of diffusion data were performed using Statistical Parametric Mapping (SPM) 8 implemented in Matlab. SPM8 instead of SPM12 was used here, this is because our preprocessing procedure was optimized and the quality of preprocessed images was validated using SPM 8 (Takeuchi et al., 2018). The methods for the preprocessing of diffusion data were described in our previous study and reproduced below (Takeuchi et al., 2019). Basically, we normalized MD, FA, gray matter segment [regional gray matter density (rGMD) map], white matter segment [regional white matter density (rWMD) map], cerebrospinal fluid (CSF) segments [regional CSF density (rCSFD) map] of diffusion images of subjects with a previously validated, modified version of DARTEL-based registration process (Takeuchi et al., 2018) method to give images with 1.5 × 1.5 × 1.5 mm^3^ voxel size. Then normalized MD images were masked by the custom mask image that is highly likely to be the gray or white matter (see Supplemental Methods for details), the normalized images were smoothed by Gaussian Kernel of 8-mm full width at half maximum (FWHM) and normalized FA images were masked by the custom mask image that is highly likely to be the white matter and then smoothed by Gaussian Kernel of 6-mm FWHM. For more details of preprocessing and how partial voluming effects were removed, see Supplementary Methods.

### Whole-Brain Statistical Analysis

We assessed rGMV, rWMV, MD, and FA associated with individual differences in family annual income and parents’ average highest educational qualifications as well as the effects of interactions between sex and family annual income and parents’ average highest educational qualifications. Statistical analyses of imaging data were performed with SPM8 (SPM8 was used because of its compatibility with the software, and see the Supplemental Methods for details). We performed two whole-brain ANCOVAs and two separate ANCOVAs (one for family annual income and one for parents’ average highest educational qualifications). This procedure of separate analyses of family income and parents’ education levels is consistent with a similar previous study (Noble et al., 2015) and based on the conceptual overlap of the two socioeconomic status measures. In addition, we did not include numerous psychological correlates (traits, cognitive functions, and states) of SES as covariates because we regarded these covariates and brain imaging properties as parallel phenomena. And like researchers do not regress out the effects of muscle power when they try to investigate association between nutrition and muscle amount, we believe that not regressing out the effects of psychological correlates of SES is appropriate for these whole-brain analyses of SES.

Analyzed using analyses of covariance for rGMV and rWMV were performed with sex, age, and either family annual income or parents’ average highest educational qualifications. ANCOVAs for MD and FA were performed with the same variables, with the exception that the total intracranial volume (TIV) that was calculated as described previously (Hashimoto et al., 2015) was added (TIV was not included as a covariate for volume analyses due to the previous studies’ findings on global effects of family SES on volume measures, in such case inclusion of global measures as covariates are improper). For the discussion, regarding how the inclusion of the global effect as a covariate is improper when there are supposed to be wide spread effects as it erases the effective findings, see our previous study (Takeuchi et al., 2010b). Analyses for rGMV and rWMV were performed in voxels for all subjects that showed a signal intensity of >0.05. Analyses for MD and FA were performed within the aforementioned gray + white matter mask and white matter mask, respectively.

For all imaging analyses (i.e., rGMV, rWMV, MD, and FA), multiple comparison correction was performed using threshold-free cluster enhancement (TFCE) (Smith and Nichols, 2009) with randomized (5,000 permutations) non-parametric testing using the TFCE toolbox^1^. We applied a threshold of FWE corrected at *P* < 0.05. We used FDR approach in behavioral analyses due to its sensitivity but used FWE approach in permutation analyses as that is the standard analysis as has been performed in our previous studies (e.g., Takeuchi et al., 2016c).

### *Post hoc* Analyses of the Associations Between the Significant Imaging Correlates and the Significant Psychological Correlates of the Family SES’s Effects

Next, we extracted the mean values of the significant imaging correlates (clusters) of the family income’s effects and investigated whether the significant psychological correlates of family income were associated with those values in the same way (directions). These associations were analyzed using ANCOVAs, with age and sex as covariates and each mean value of the family income’s effects’ significant imaging correlates (clusters) as a dependent variable. To model these analyses, both the age and one of the significant psychological correlates of family SES were used as covariates. Sex was a fixed factor, and the interaction between sex and one of the significant psychological correlates of the family SES was included. Then, we also performed the same analyses on the significant correlates of the parents’ educational qualifications.

Here, because there were a number of significant clusters and psychological correlates of family SES (we applied *p* < 0.01, uncorrected, for the statistical threshold), only the results showing association with the imaging correlates of family SES in the same direction were noted. Moreover, these analyses present the difficulties of double dipping procedures (Kriegeskorte et al., 2009); therefore, all results are exploratory and supplemental.

## Results

### Basic Data

**FIGURE 1:**
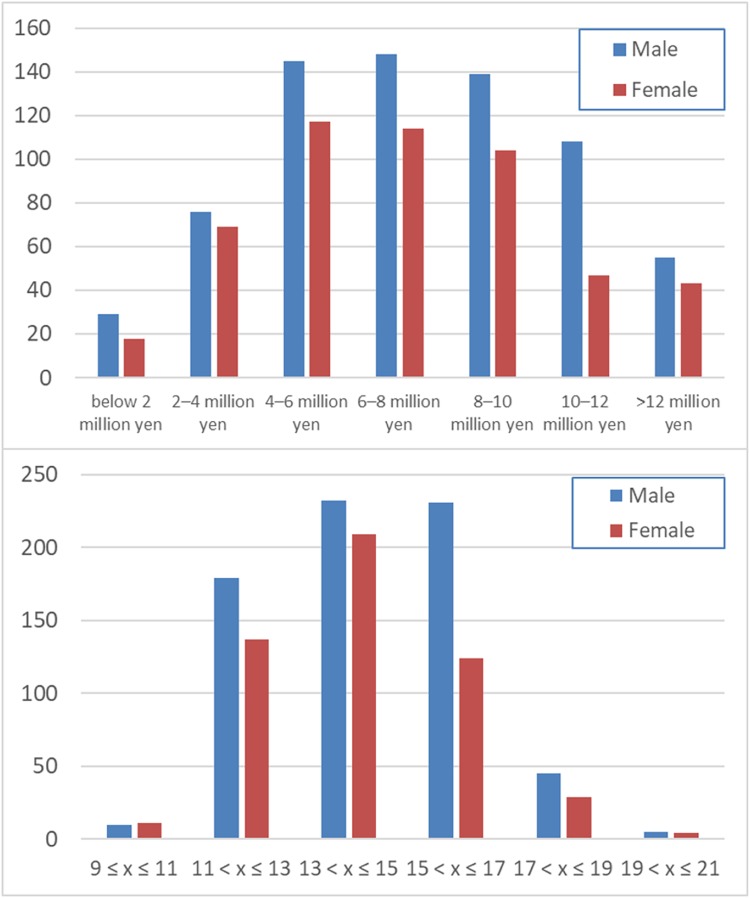
Distribution of the family annual income (upper panel) and the average of the parents’ highest educational qualifications (lower panel).

The average (and SD) age, RAPM score, family annual income, and parents’ average highest educational qualifications for men and women are shown in Table 1. The distributions of family annual income and parents’ average highest educational qualifications are presented in Figure 1. Note the difference of the number of subjects in each sex would not bias the results of interaction analyses involving sex. And since our study deals with the sex interaction analysis (meaning the difference of the correlation between men and women), the subtle difference of age (i.e., 0.2 years) or other characteristics between sex are generally not supposed to substantially affect the results of interaction analyses as the most of the range of such characteristics overlap between sex and among such mostly overlapped range of various characteristics, we see the sex difference of the correlation between family SES and other variables.

**Table 1:** Demographic variables of the study participants.

	Male (*N* = 702)		Female (*N* = 514)	
	
Measure	Mean	*SD*	Mean	*SD*
Age	20.80	1.89	20.60	1.60
RAPM	28.79	3.86	28.11	3.81
Family annual income^∗^	4.19	1.58	4.04	1.55
Parents’ average educational qualification	14.75	1.87	14.51	1.85


*^∗^Family annual income was classified as follows: 1, annual income below 2 million yen; 2, 2–4 million yen; 3, 4–6 million yen; 4, 6–8 million yen; 5, 8–10 million yen; 6, 10–12 million yen; 7, >12 million yen.*

Family annual income and parents’ average highest educational qualifications showed a robust significant substantial correlation (simple correlation, *r* = 0.412, *P* = 5.91 × 10^-51^), supporting the by far the most standard separate modeling of family annual income and parents’ average highest educational qualifications.

### Psychological Analyses of Effects of Family Annual Income

The effects of family annual income on a wide range of psychological variables were investigated. After correcting for multiple comparisons, ANCOVAs revealed that there were significant main positive effects (regardless of sex) of family annual income on the total score of the Emotional Intelligence Scale, average score of WHOQOL, score of the competitive achievement motivation Scale, score of self-esteem, score of need for uniqueness, nationalism, extraversion, and positive life events as well as significant negative effects on the score of pessimism, score of the UCLA Loneliness Scale, and trait anxiety score of STAI. There was a significant effect of the interaction between sex and family annual income on the score of SESRA-S. This interaction had a negative effect on family annual income in males and a positive effect on family income in females. For statistical values, see Table 2.

**Table 2:** Main effects of family annual income as well as effects of interaction between sex and family annual income on psychological measures.

	N male	N female	Correlation coefficient male	Correlation coefficient female	Main effect *F*-value	Main effect *P*-value, uncorrected	Main effect, *P*-value, FDR^1^	Interaction effect *F*-value	Interaction effect *P*-value uncorrected	Interaction effect *P*-value FDR
**Cognitive functions**										
RAPM^2^(intelligence, non-verbal reasoning)	699	512	0.022	0.032	0.846	0.358	0.482	0.032	0.858	0.712
TBIT^3^(intelligence)	632	457	0.038	0.036	1.33	0.249	0.372	0.005	0.942	0.727
Reading comprehension	608	422	-0.004	-0.006	0.014	0.907	0.719	0.001	0.974	0.736
S-A creativity test	700	512	0.069	-0.028	0.414	0.52	0.57	2.945	0.086	0.181
Digit span	697	509	-0.03	0.007	0.177	0.674	0.645	0.413	0.521	0.57
**Traits and states**										
Empathizing	700	512	0.039	0.099	5.08	0.024	0.085	0.954	0.329	0.461
Systemizing	700	512	0.02	0.126	5.188	0.023	0.084	2.473	0.116	0.226
Emotional Intelligence Scale	697	512	0.091	0.121	12.482	4.26 × 10^-4^	0.005	0.142	0.706	0.655
GHQ30^4^	696	511	-0.068	-0.006	1.524	0.217	0.338	1.119	0.29	0.423
WHOQOL-26	697	512	0.148	0.126	21.719	4.00 × 10^-6^	4.6 × 10^-4^	0.271	0.603	0.596
Critical thinking disposition	698	512	4.62 × 10^-4^	0.089	1.812	0.179	0.293	1.872	0.172	0.293
Cognitive reflectivity–Impulsiveness	697	512	0.007	-0.045	0.488	0.485	0.552	0.843	0.359	0.482
Need for Cognition	698	512	0.011	0.063	1.345	0.246	0.372	0.618	0.432	0.54
Self-fulfillment Achievement Motivation	698	512	0.012	0.033	0.506	0.477	0.552	0.102	0.75	0.678
Competitive Achievement Motivation	698	512	0.037	0.106	6.704	0.01	0.045	1.832	0.176	0.293
Self-esteem	698	512	0.097	0.098	10.767	0.001	0.011	8.6 × 10^-5^	0.993	0.736
Need for uniqueness	697	512	0.042	0.148	9.917	0.002	0.014	2.829	0.093	0.187
Patriotism	697	512	0.085	-0.015	1.583	0.209	0.329	2.973	0.085	0.181
Nationalism	697	512	0.114	0.099	13.25	2.84 × 10^-4^	0.005	0.162	0.688	0.645
Optimism	697	512	0.068	0.039	3.024	0.082	0.181	0.271	0.603	0.596
Pessimism	697	512	-0.063	-0.113	8.77	0.003	0.022	0.631	0.427	0.54
Self-efficacy	697	511	0.061	0.081	5.159	0.023	0.084	0.017	0.895	0.719
UCLA Loneliness Scale	698	512	-0.069	-0.1	7.839	0.005	0.028	0.168	0.682	0.645
CIS-20^5^ (fatigue)	696	512	-0.113	-0.033	5.767	0.016	0.065	2.05	0.152	0.283
Beck Depression Inventory	640	475	-0.072	-0.102	8.339	0.004	0.023	0.333	0.564	0.595
STAI^6^_state (anxiety state)	640	475	-0.064	-0.08	5.39	0.02	0.078	0.055	0.815	0.694
STAI_trait (anxiety trait)	640	475	-0.087	-0.125	11.944	0.001	0.007	0.572	0.449	0.545
POMS-TMD^7^ (mood disturbance)	688	508	-0.102	-0.074	8.79	0.003	0.022	0.097	0.755	0.678
Neuroticism	699	512	-0.04	-0.095	4.792	0.029	0.089	0.779	0.377	0.499
Extraversion	699	512	0.092	0.083	8.417	0.004	0.023	0.083	0.774	0.69
Openness	699	512	0.023	0.034	0.924	0.337	0.466	0.037	0.848	0.712
Agreeableness	699	512	-0.038	-0.028	1.653	0.199	0.322	0.006	0.936	0.727
Conscientiousness	699	512	0.022	0.073	2.352	0.125	0.24	0.64	0.424	0.54
Jealousy	643	475	-0.052	-0.081	4.044	0.045	0.116	0.266	0.606	0.596
Self-Preoccupation	643	475	-0.042	-0.083	3.674	0.056	0.134	0.491	0.484	0.552
External-Preoccupation	643	475	-0.019	0.095	1.834	0.176	0.293	3.787	0.052	0.133
SESRA-S^8^ (sex role attitude)	643	475	-0.069	0.095	0.1	0.752	0.678	7.206	0.007	0.037
**Life event**										
Negative life event	700	512	0.003	0.022	0.419	0.517	0.57	0.215	0.643	0.627
Positive life event	700	512	0.043	0.123	8.383	0.004	0.023	1.978	0.16	0.283


*^*1*^False discovery rate. ^*2*^Raven’s Advanced Progressive Matrices. ^*3*^Tanaka B-type intelligence test. ^*4*^General Health Questionnaire 30. ^*5*^Checklist individual strength. ^*6*^State-Trait Anxiety Inventory. ^*7*^Profile of Mood States (POMS). Total mood disturbance. ^*8*^Egalitarian Sex Role Attitudes-Short Form.*

### Psychological Analyses of Effects of Parents’ Average Highest Educational Qualifications

The effects of parents’ average highest educational qualifications on a wide range of psychological variables were investigated. After correcting for multiple comparisons, ANCOVAs revealed that there were significant main positive effects (regardless of sex) of parents’ average highest educational qualifications on the scores of intelligence tasks, including the speeded task (TBIT), S-A creativity test (creativity measured by divergent thinking), systemizing, WHOQOL26 (QOL measure), and the measure for the need for cognition and openness as well as significant main negative effects on the score of CIS (measure of chronic fatigue) and STAI’s state and trait measures (measure of state and trait of anxiety). For statistical values, see Table 3.

**Table 3:** Main Effects of parents’ average highest educational qualifications as well as effects of interaction between sex and parents’ average highest educational qualifications on psychological measures.

	N male	N female	Correlation coefficient male	Correlation coefficient female	Main effect *F*-value	Main effect *P*-value, uncorrected	Main effect, *P*-value, FDR	Interaction effect *F*-value	Interaction effect *P*-value uncorrected	Interaction effect *P*-value FDR
**Cognitive functions**										
RAPM (intelligence, non-verbal reasoning)	701	514	0.088	0.054	6	0.014	0.062	0.353	0.553	0.588
TBIT (intelligence)	634	459	0.114	0.117	13.97	1.95 × 10^-4^	0.004	0.006	0.939	0.727
Reading comprehension	608	423	0.048	0.03	1.583	0.209	0.329	0.069	0.793	0.691
S-A creativity test	702	514	0.153	0.098	18.603	1.7 × 10^-5^	0.001	0.986	0.321	0.461
Digit span	699	511	0.011	0.021	0.274	0.601	0.596	0.02	0.889	0.719
**Traits and states**										
Empathizing	702	514	0.004	0.033	0.318	0.573	0.596	0.394	0.531	0.576
Systemizing	702	514	0.054	0.165	12.678	3.84 × 10^-4^	0.005	2.978	0.085	0.181
Emotional Intelligence Scale	699	514	0.072	0.071	5.834	0.016	0.065	4.16 × 10^-4^	0.984	0.736
GHQ30	698	513	-0.088	0.005	1.999	0.158	0.283	2.531	0.112	0.222
WHOQOL-26	699	514	0.12	0.068	10.369	0.001	0.012	0.837	0.361	0.482
Critical thinking disposition	700	514	0.052	0.175	14.553	1.43 × 10^-4^	0.004	4.446	0.035	0.101
Cognitive reflectivity–Impulsiveness	699	514	-0.024	0.072	0.762	0.383	0.5	2.855	0.091	0.187
Need for Cognition	700	514	0.054	0.12	8.674	0.003	0.022	1.329	0.249	0.372
Self-fulfillment Achievement Motivation	700	514	0.045	0.084	4.887	0.027	0.089	0.558	0.455	0.545
Competitive Achievement Motivation	700	514	-0.037	-0.046	2.027	0.155	0.283	0.06	0.806	0.694
Self-esteem	700	514	0.059	0.071	4.835	0.028	0.089	0.075	0.784	0.691
Need for uniqueness	699	514	0.135	0.082	13.964	1.95 × 10^-4^	0.004	0.961	0.327	0.461
Patriotism	699	514	0.044	-0.066	0.1	0.752	0.678	3.503	0.062	0.141
Nationalism	699	514	0.061	-0.062	0.003	0.96	0.736	4.334	0.038	0.103
Optimism	699	514	0.08	0.04	4.111	0.043	0.115	0.371	0.543	0.583
Pessimism	699	514	-0.058	-0.047	3.161	0.076	0.171	0.031	0.861	0.712
Self-efficacy	699	513	0.056	0.059	3.666	0.056	0.134	0.014	0.906	0.719
UCLA Loneliness Scale	700	514	-0.041	-0.04	1.869	0.172	0.293	1.32 × 10^-4^	0.991	0.736
CIS-20 (fatigue)	698	514	-0.082	-0.07	6.677	0.01	0.045	0.015	0.903	0.719
Beck Depression Inventory	641	476	-0.062	-0.073	4.976	0.026	0.088	0.069	0.792	0.691
STAI_state (anxiety state)	641	476	-0.088	-0.078	7.418	0.007	0.034	0.023	0.88	0.719
STAI_trait (anxiety trait)	641	476	-0.088	-0.109	10.467	0.001	0.012	0.291	0.589	0.596
POMS-TMD (mood disturbance)	690	510	-0.095	-0.022	3.691	0.055	0.134	1.198	0.274	0.404
Neuroticism	701	514	-0.048	-0.08	4.608	0.032	0.097	0.478	0.49	0.552
Extraversion	701	514	0.095	0.053	6.302	0.012	0.054	0.483	0.487	0.552
Openness	701	514	0.097	0.125	14.661	1.35 × 10^-4^	0.004	0.305	0.581	0.596
Agreeableness	701	514	-0.022	-0.023	0.721	0.396	0.512	0.007	0.935	0.727
Conscientiousness	701	514	-0.018	0.023	0.001	0.976	0.736	0.57	0.451	0.545
Jealousy	644	476	-0.078	-0.053	4.34	0.037	0.103	0.055	0.815	0.694
Self-Preoccupation	644	476	-0.025	-0.005	0.159	0.69	0.645	0.042	0.838	0.708
External-Preoccupation	644	476	-0.036	0.075	0.559	0.455	0.545	3.507	0.061	0.141
SESRA-S (sex role attitude)	644	476	-0.027	0.063	0.279	0.597	0.596	2.164	0.142	0.267
**Life event**										
Negative life event	702	514	0.014	0.021	0.507	0.477	0.552	1.35 × 10^-6^	0.999	0.736
Positive life event	702	514	0.043	0.123	4.447	0.035	0.101	0.169	0.681	0.645


### Main Effects of Family Annual Income on Imaging Measures Regardless of Sex

**FIGURE 2:**
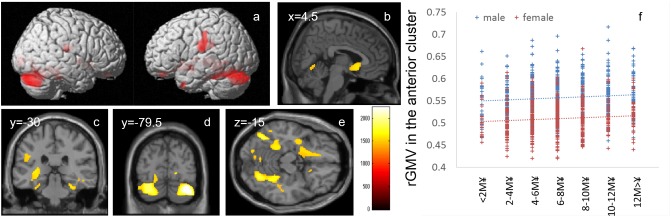
Positive main effects of family annual income on rGMV. **(a–e)** The results shown were obtained using a threshold of threshold-free cluster enhancement (TFCE) of *P* < 0.05 based on 5000 permutations. The results were corrected at the whole-brain level. **(b–e)** Regions with significant correlations are overlaid on a “single subject” T1 image of SPM8. The color represents the strength of the TFCE value. Significant positive main effects of family annual income on family income were mainly observed in **(a)** the bilateral perisylvian areas, **(b)** subgenual cingulate gyrus, **(c)** bilateral hippocampus, **(d)** bilateral cerebellum, and **(e)** bilateral fusiform gyrus and occipital areas. **(e)** A scatter plot with trend lines depicting correlations between mean rGMV in the significant cluster of the anterior brain areas in males (blue) and females (red). **(f)** A scatter plot with trend lines depicting correlations with mean rGMV for the largest significant cluster in males (blue) and females (red).

Whole-brain ANCOVA revealed an overall positive main effect (regardless of sex) of family annual income on rGMV in the bilateral cerebellum, bilateral calcarine cortex, bilateral lingual gyrus, bilateral fusiform gyrus, bilateral medial temporal lobe areas (parahippocampal gyrus, hippocampus, and amygdala), bilateral perisylvian areas, left temporal pole, areas in the basal ganglia, insula and orbital frontal areas, and the subgenual cingulate gyrus (Figure 2). For complete information on brain areas and statistical values, see Table 4.

**Table 4:** Brain regions exhibiting significant main positive effects of family income on rGMV.

	Included gray matter areas (number of significant voxels in the left and right side of each anatomical area)^∗^	*x*	*y*	*Z*	TFCE-value	Corrected *P*-value (FWE)	Cluster size (voxel)
1	Calcarine Cortex (L:7, R:320)/Fusiform gyrus (R:1300)/Lingual gyrus (R:426)/Parahippocampal gyrus (R:71)/Inferior temporal gyrus (R:20)/Middle temporal gyrus (R:2)/Cerebellum (R:5284)/	37.5	-81	-33	2238.828	0.011	7629
2	Fusiform gyrus (L:1255)/Heschl gyrus (L:5)/Hippocampus (L:312)/Insula (L:268)/Lingual gyrus (L:303, R:21)/Inferior occipital lobe (L:137)/Parahippocampal gyrus (L:379)/Inferior parietal lobule (L:47)/Postcentral gyrus (L:817)/Putamen (L:13)/Rolandic operculum (L:443)/Supramarginal gyrus (L:628)/Inferior temporal gyrus (L:578)/Middle temporal gyrus (L:92)/Superior temporal gyrus (L:103)/Thalamus (L:3710, R:3)/	-46.5	-69	-22.5	1909.474	0.0202	10585
3	Amygdala (L:62)/Caudate (L:365, R:339)/Superior frontal orbital area (L:153)/Hippocampus (L:1)/Insula (L:205)/Pallidum (L:102, R:12)/Putamen (L:333, R:4)/Rectus gyrus (L:456, R:63)/Rolandic operculum (L:2)/Superior temporal gyrus (L:10)/	-13.5	16.5	-10.5	1593.939	0.037	3505
4	Inferior temporal gyrus (L:82)/Temporal pole (L:66)/	-42	10.5	-48	1483.472	0.0448	348
5	Heschl gyrus (R:7)/Insula (R:1)/Rolandic operculum (R:338)/Supramarginal gyrus (R:58)/Superior temporal gyrus (R:36)/	54	-22.5	18	1475.474	0.045	448
6	Insula (R:113)/Putamen (R:11)/	40.5	-1.5	0	1442.859	0.048	193
7	Parahippocampal gyrus (R:2)	9	-6	-19.5	1436.221	0.0482	76


*^∗^Labelings of the anatomical regions of gray matter were based on the WFU PickAtlas Tool (http://www.fmri.wfubmc.edu/cms/software#PickAtlas/) (Maldjian et al., 2003, 2004) and on the PickAtlas automated anatomical labeling atlas option (Tzourio-Mazoyer et al., 2002). Temporal pole areas included all subregions in the areas of this atlas.*

Whole-brain ANCOVAs revealed that there were no significant main effects of family income (regardless of sex) on rWMV, FA, and MD.

### Effects of Interaction Between Sex and Family Annual Income on Imaging Measures

**FIGURE 3:**
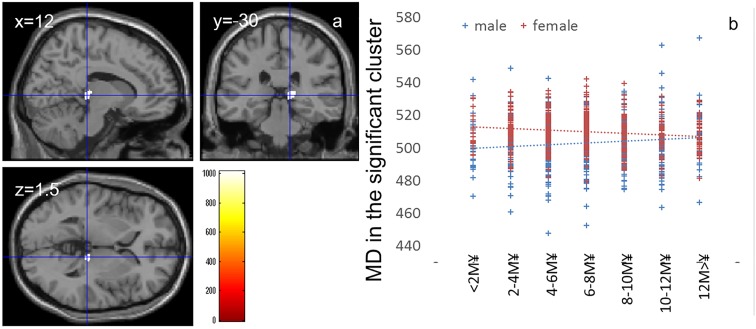
Effects of interaction between sex and family annual income on MD that show a positive correlation in males and negative correlation in females. **(a)** The results shown were obtained using a threshold of threshold-free cluster enhancement (TFCE) of *P* < 0.05, based on 5000 permutations. The results were corrected at the whole brain level. Regions with significant correlations are overlaid on a “single subject” T1 image of SPM8. The color represents the strength of the TFCE value. Significant effects were observed in the thalamus. **(b)** A scatter plot with trend lines depicting correlations between mean MD for significant clusters of males (blue) and females (red).

Whole-brain ANCOVA revealed effects of the interaction between sex and family annual income on MD in the thalamus [Figure 3, *x*, *y*, *z* = 12, -30, 1.5, TFCE value = 1012.64, *P* = 0.042, corrected and 174 voxels with the threshold of *P* < 0.05 corrected for multiple comparisons (permutation using TFCE values, FWE)]. Note the interaction is formed by both of tendencies of positive correlation in males and negative correlation in females as seen in Figure 3.

Whole-brain ANCOVAs revealed that there were no significant effects of interaction between sex and family annual income on rGMV, rWMV, and FA.

### Main Effects of Parents’ Average Highest Educational Qualifications on Imaging Measures Regardless of Sex

**FIGURE 4:**
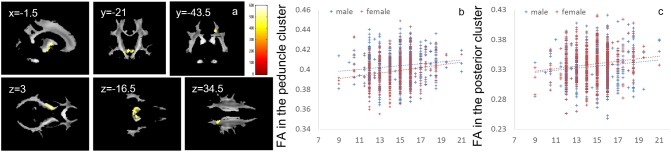
Positive main effects of parents’ average highest educational qualifications on FA. **(a)** The results shown were obtained using a threshold of threshold-free cluster enhancement (TFCE) of *P* < 0.05, based on 5000 permutations. The results were corrected at the whole brain level. Regions of correlation were overlaid on mean preprocessed, but not smoothed, FA images of a subset of participants. The color represents the strength of the TFCE value. Significant effects were observed near the peduncle and in posterior white matter areas. **(b–c)** Scatter plots with trend lines depicting correlations of mean FA for significant clusters in males (blue) and females (red) in the areas around the internal capsule and peduncle **(b)** and the white matter area in the parietal cortex **(c)**.

Whole-brain ANCOVA revealed an overall positive main effect (regardless of sex) of parents’ average highest educational qualifications on FA in the white matter areas of the cerebral peduncle and internal capsule as well as white matter areas of the posterior brain, including part of the splenium of the corpus callosum and right posterior corona radiata (Figure 4). For complete information on brain areas and statistical values, see Table 5.

**Table 5:** Brain regions exhibiting significant main positive effects of parents’ average educational qualification on FA.

	Included large bundles^∗^ (number of significant voxels in left and right side of each anatomical area)	*x*	*y*	*z*	TFCE value	Corrected *p*-value (FWE)	Cluster size (voxel)
1	Superior cerebellar peduncle (L:1)/Cerebral peduncle (L:91, R:57)/Anterior limb of internal capsule (L:50)/Posterior limb of internal capsule (L:155)/Retrolenticular part of internal capsule (L:1)/	-1.5	-25.5	-13.5	598.9525	0.0086	729
2	Splenium of corpus callosum (13)/Posterior corona radiata (R:99)/	18	-43.5	34.5	517.5639	0.0168	170
3	None	7.5	-7.5	-15	407.5656	0.0424	5
4	None	-24	-4.5	13.5	393.8078	0.0494	2


*^∗^The anatomical labels and significant clusters of major white matter fibers were determined using the ICBM DTI-81 Atlas (http://www.bmap.ucla.edu/portfolio/atlases/ICBM_DTI-81_Atlas/).*

Whole-brain ANCOVAs revealed that there were no significant effects of parents’ average highest educational qualifications on rGMV, rWMV, and MD.

### Effects of Interaction Between Sex and Parents’ Average Highest Educational Qualifications on Imaging Measures

**FIGURE 5:**
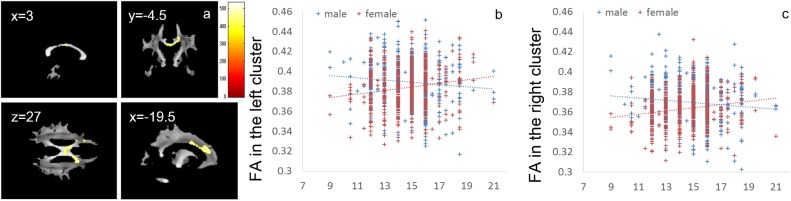
Effects of interaction between sex and parents’ average highest educational qualifications on FA that are moderated by positive correlation in females and negative correlation in males. **(a)** The results shown were obtained using a threshold of threshold-free cluster enhancement (TFCE) of *P* < 0.05, based on 5000 permutations. The results were corrected at the whole brain level. Regions of correlation were overlaid on mean preprocessed, but not smoothed, FA images of a subset of participants. The color represents the strength of the TFCE value. Significant effects were observed in areas around the body and genu of the corpus callosum and the bilateral corona radiata. **(b–c)** Scatter plots with trend lines depicting correlations with mean FA for significant clusters in males (blue) and females (red) in white matter areas in the left hemisphere **(b)** and right hemisphere **(c)**.

Whole-brain ANCOVA revealed effects of interaction between sex and parents’ average highest educational qualifications on FA in white matter areas of the genu and body of the corpus callosum and bilateral corona radiata (Figure 5). For complete information on brain areas and statistical values, see Table 6. Note the interaction is formed by both of tendencies of negative correlation in males and positive correlation in females as seen in Figure 5. The areas of significant interaction did not overlap with the areas of the main effects.

**Table 6:** Brain regions exhibiting significant effects of interaction between parents’ average educational qualification and sex (that are moderated by positive correlation in females and negative correlation in males) on FA.

	Included large bundles^∗^ (number of significant voxels in left and right side of each anatomical area)	*x*	*y*	*z*	TFCE value	Corrected *p* value (FWE)	Cluster size (voxel)
1	Genu of corpus callosum (18)/Body of corpus callosum (283)/Anterior corona radiata (L:189)/Superior corona radiata (L:68)/	–18	3	31.5	534.0439	0.0126	618
2	Genu of corpus callosum (14)/Body of corpus callosum (304)/Anterior corona radiata (R:213)/Superior corona radiata (R:175)	19.5	16.5	25.5	499.8084	0.0164	801


*^∗^The anatomical labels and significant clusters of major white matter fibers were determined using the ICBM DTI-81 Atlas (http://www.loni.ucla.edu/).*

**FIGURE 6:**
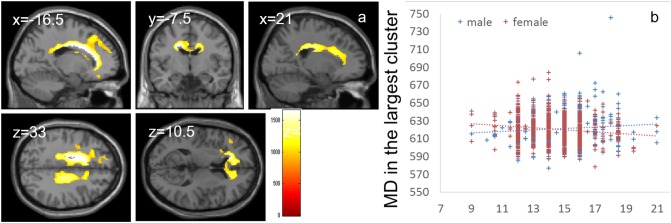
Effects of interaction between sex and parents’ average highest educational qualifications on MD that are moderated by positive correlation in males and negative correlation in females. **(a)** The results shown were obtained using a threshold of threshold-free cluster enhancement (TFCE) of *P* < 0.05, based on 5000 permutations. The results were corrected at the whole brain level. Regions with significant correlations are overlaid on a “single subject” T1 image of SPM8. The color represents the strength of the TFCE value. Significant effects were observed in areas around the body and genu of the corpus callosum, bilateral corona radiata, caudate, prefrontal areas, and insula. **(b)** A scatter plot with trend lines depicting correlations with mean MD for the largest significant cluster in males (blue) and females (red).

Whole-brain ANCOVA revealed effects of interaction between sex and parents’ average highest educational qualifications on MD in the white matter areas of the body and genu of the corpus callosum as well as the bilateral corona radiata, caudate, prefrontal areas, and insula (Figure 6). For complete information on brain areas and statistical values, see Table 7. Note the interaction is formed by both of tendencies of positive correlation in males and negative correlation in females as seen in Figure 6.

**Table 7:** Brain regions exhibiting significant effects of interaction between parents’ average educational duration and sex (that are moderated by positive correlation in males and negative correlations in females) on MD.

Included gray matter areas^∗^(number of significant voxels in left and right side of each anatomical area)	Included large bundles^∗∗^ (number of significant voxels in left and right side of each anatomical area)	*x*	*y*	*z*	TFCE value	Corrected *p*-value (FWE)	Cluster size (voxel)
Caudate (L:129, R:64)/Anterior cingulum (L:814, R:1135)/Middle cingulum (L:642, R:420)/Posterior cingulum (R:3)/Inferior frontal orbital area (L:40)/Middle frontal medial area (R:33)/Middle frontal orbital area (L:121)/Middle frontal other areas (L:612)/Superior frontal medial area (L:390, R:23)/Superior frontal orbital area (L:63)/Superior frontal other areas (L:729)/Insula (L:111)/Supplemental motor area (L:1)/	Genu of corpus callosum (1478)/Body of corpus callosum (1993)/Splenium of corpus callosum (551)/Anterior limb of internal capsule (L:30)/Anterior corona radiata (L:1551, R:847)/Superior corona radiata (L:951, R:750)/Posterior corona radiata (L:123, R:383)/External capsule (L:10)/Cingulum (L:458, R:234)/Superior longitudinal fasciculus (R:1)/Superior fronto-occipital fasciculus (L:42, R:5)/Tapatum (L:15, R:20)	–16.5	3	33	1659.942	0.0072	14756
Inferior frontal operculum (L:38)/Inferior frontal triangular (L:354)/Middle frontal other areas (L:17)/Precentral gyrus (L:162)/	None	–34.5	1.5	36	984.6044	0.04	619
Inferior frontal triangular (L:11)/Middle frontal other areas (L:9)	None	–43.5	34.5	27	911.0997	0.0494	20


*^∗^Labelings of the anatomical regions of gray matter were based on the WFU PickAtlas Tool (http://www.fmri.wfubmc.edu/cms/software#PickAtlas/) (Maldjian et al., 2003, 2004) and on the PickAtlas automated anatomical labeling atlas option (Tzourio-Mazoyer et al., 2002). Temporal pole areas included all subregions in the areas of this atlas. ^∗∗^The anatomical labels and significant clusters of major white matter fibers were determined using the ICBM DTI-81 Atlas (http://www.loni.ucla.edu/).*

Whole-brain ANCOVAs revealed that there were no significant effects of interaction between sex and parents’ average highest educational qualifications on rGMV and rWMV.

### *Post hoc* Analyses of the Associations Between the Significant Imaging Correlates and the Significant Psychological Correlates of Family SES’s Main Effects

Regarding the *post hoc* analyses of family income, the *post hoc* analyses showed: (1) a significantly negative main effect of pessimism on the mean rGMV values of the significant cluster 1 (Table 2), which mainly involves the right calcarine cortex, right fusiform gyrus, right parahippocampal gyrus, and right cerebellum (*p* = 0.006); (2) a significantly negative main effect of pessimism on the mean rGMV values of the significant cluster 2 (Table 4), which mainly involves the left fusiform gyrus, left hippocampus and parahippocampal gyrus, left insula, left lingual gyrus, left occipital lobe, left parietal lobe, and perisylvian areas and the left thalamus (*p* = 0.006); (3) a significantly positive main effect of the competitive achievement motivation score on the mean rGMV values of the significant cluster 5 (Table 4), which mainly involves the right insula, right Rolandic operculum, right supramarginal gyrus, and right superior temporal gyrus (*p* = 0.008); (4) a significantly negative main effect of the Beck Depression Inventory score on the mean rGMV values of the significant cluster 5 (Table 4), which involves the right insula, right Rolandic operculum, right supramarginal gyrus, and right superior temporal gyrus (*p* = 0.008); and (5) a significantly positive main effect of the positive life events score on the mean rGMV values of the significant cluster 7 (Table 4), which involves the right parahippocampal gyrus (*p* = 0.010).

Regarding the *post hoc* analyses of the parents’ educational qualification, under the applied criteria, no significant association was found between the significant imaging correlates of the parents’ educational qualification and psychological correlates of the parents’ educational qualification in the specified direction. However, although in this study the parents’ educational qualifications were not significantly associated with rGMV in whole brain analyses, the scores of intelligence tasks (TBIT; which comprise a significant psychological correlate of the parents’ educational qualification’s main effect) showed significant positive main effects (regardless of sex) on the mean rGMV values of the significant cluster 3 (Table 4; significant correlates of main effects of family income; *p* = 0.003) and on the mean rGMV of the significant cluster 7 (Table 4; *p* = 0.005).

## Discussion

In this study, we newly investigated the effects of family SES on MD as well as the effects of interaction between family SES and sex, particularly in terms of neural correlates. In contrast to our hypothesis, there were no significant main effects (regardless of sex) of family SES on MD. However, our first novel findings were that partly consistent with our hypothesis, there were significant effects of interaction between sex and parents’ educational qualification on MD and FA in the body of the corpus callosum as well as in the white matter areas between the anterior cingulate cortex and lateral prefrontal cortex. Our second novel findings were, there was a significant effect between sex and family income on MD was in the thalamus. Consistent with previous studies, higher family income was associated with larger rGMV in areas related to effects, language-related areas, in both sexes together with other areas such as the visual cortex. Furthermore, higher educational qualification of parents was associated with greater FA in the white matter areas connecting cortical and subcortical areas.

Additional psychological analyses showed that greater family annual income was associated with a wide range of psychological measures related to positive effects and experiences (greater QOL, self-esteem, and experiences of positive life events as well as lesser pessimism, loneliness, and trait anxiety) and traits that are supported by or support positive effects (greater emotional intelligence, extraversion, and need for uniqueness). These findings are generally consistent with the pattern reported in previous studies (lesser psychological distress, lesser stress, and greater well-being) (Ursache and Noble, 2016a). In contrast to previous studies, we found that family annual income was not associated with cognitive function (Ursache and Noble, 2016a). This may be because we focused on college students and the educational qualification of subjects was generally high. Among individuals in this study population, family income may have little association with cognitive function. Interestingly, family income was associated with greater nationalism and competitive achievement motivation, which is a third novel finding. The reason for this association is not clear; however, we have previously shown greater nationalism is associated with a feeling of superiority to others (Takeuchi et al., 2016b), and the data in the present sample also showed a positive correlation between competitive achievement motivation and a feeling of superiority to others. This kind of feeling caused by greater family income may lead to nationalism and competitive achievement motivation. Our results suggest that greater annual family income is associated with a greater stereotype regarding the sex role in males but a smaller stereotype regarding the sex role in females. This finding is congruent with the previous report that high SES females are more likely than low SES females to choose male-dominated occupations (Hannah and Kahn, 1989) and may reflect that regardless of sex, subjects hold beliefs that allow them to work. On the other hand, higher educational qualification of parents was positively associated with greater cognitive functioning (intelligence and creativity measured by divergent thinking) and greater traits and states related to more positive effects and experiences (greater QOL, lesser chronic fatigue, and lesser state and trait anxiety), which is consistent with the findings of previous studies (Ursache and Noble, 2016a). The present finding, which was not reported in the previous review (Ursache and Noble, 2016a), was that higher educational qualification of parents was associated with increase in multiple traits that facilitate more academic thoughts, such as systemizing (drive to analyze a system) (Baron-Cohen et al., 2005), need for cognition (tendency for an individual to engage in and enjoy thinking) (Kouyama and Fujiwara, 1991), and openness (which includes intellectual curiosity and ideas) (Costa and MacCrae, 1992). This effect may be due to the parents’ educational qualification or genetics (parents with greater familiarity with academic issues tend to have children with the same characteristic), but this speculation cannot be validated with a cross-sectional study.

Higher family income was associated with greater rGMV in (a) areas involved in language, such as the cerebellum, fusiform gyrus, lingual gyrus, and perisylvian areas, and (b) areas involved in effects, mood, and stress, such as the insula, the hippocampus, amygdala, and the subgenual cingulate gyrus, and its surrounding area (although the results are widespread and include other sparse areas such as areas in the visual cortex). Language areas, medial temporal lobe structures, and the prefrontal cortex are areas associated with family SES, and the positive effects of family income on rGMV are consistent with previous findings (Ursache and Noble, 2016a). The fusiform gyrus is associated with visual word recognition (Price, 2000). The perisylvian areas are consistently activated during language processing, and cortical structures in this area play key roles in various language-related processing, such as phonological processing, syntactic processing, and articulatory processing (Price, 2000). The cerebellum is also involved in articulatory processes (Baddeley, 2003). Larger rGMV in these areas may underlie the associations between higher family SES and a wide range of greater language-related competence (Ursache and Noble, 2016a). The prefrontal cortex, particularly the medial prefrontal areas, hippocampus, and amygdala, is most affected by stress (Takeuchi and Kawashima, 2016). A previous review suggested that lower SES leads to greater stress, which in turn impacts the function and structure of these areas (Ursache and Noble, 2016a). Dysfunction of the amygdala caused by stress has been suggested to lead to dysfunction of social and emotional processing (Ursache and Noble, 2016a). Particularly, the subgenual cingulate gyrus is most consistently affected by depression and plays a key role in emotion regulation (Drevets et al., 2008). Greater rGMV in these areas may underlie the associations between higher family SES and a wide range of positive effects and self-regulatory behaviors found in this study and previous studies (Ursache and Noble, 2016a). Partly consistent with this notion, our exploratory supplemental analyses showed that (1) lower pessimism, which may decrease the personal subjective stress as well as (2) more experience of positive life events, is associated with greater rGMV of the significant clusters involving the hippocampus and contingent areas. Both these psychological characteristics were associated with a greater family income. Furthermore, the insula receives inputs from a wide range of areas and is involved in a wide range of emotions (Nagai et al., 2007). We have previously shown that the insula’s regional gray matter structure is associated with greater competitive achievement motivation (Takeuchi et al., 2014b), and a previous review pointed out that rGMV reduction in this area is associated with depression and a wide range of psychiatric disorders (Nagai et al., 2007). Partly consistent with this notion, our exploratory supplemental analyses show that both a lower level of depression and a higher competitive achievement motivation are associated with greater rGMV of the significant clusters involving the insula. Both those psychological characteristics were associated with a higher family income.

The lack of significant associations between brain volume measures and parents’ average highest educational qualifications may be due to the complex associations between neural plasticity and rGMV. Previous studies on the associations between parents’ educational qualification and measures of gray matter are inconsistent. Studies have generally shown positive associations between family SES and measures related to gray matter volume (Ursache and Noble, 2016a). However, a few studies in young adults have not shown this association. Kong et al. (2015) have revealed that a mother’s educational qualification is negatively correlated with rGMV in the medial prefrontal cortex in young adults. Yang et al. (2016) have also shown a negative relationship between family SES and rGMV in the medial prefrontal cortex. Another recent study has investigated the associations between parents’ educational qualification and cortical thickness and surface area in a wide developmental age range (Piccolo et al., 2016). This study has revealed that children of parents with high educational qualification show greater developmental cortical thinning in the later phase of development and as a result, it seems, while during the age of elementary school and junior high school, children of parents with high education level show greater cortical thickness, that difference becomes unclear by the age of the young adults (Piccolo et al., 2016). The reason for this phenomenon is not clear; however, the authors have suggested that a low level of stimulation from environments in the children of parents with low educational qualification leads to earlier pruning or a shorter window for sensitive developmental periods which are counterproductive (Piccolo et al., 2016). The mean age of participants in the present study was approximately 21 years (young adults), an age that may make the associations between rGMV and parents’ educational qualification unclear according to this observation (Piccolo et al., 2016).

There were positive correlations between FA and parents’ average highest educational qualifications in (a) the internal capsule, which connects the midbrain and extensive areas of the cortex and underlies the brain’s extensive information processing, and (b) the splenium of the corpus callosum and right posterior corona radiata, which underlies higher-order information processing. The internal capsule consists of white matter fibers that connect the midbrain and brainstem as well as extensive areas of the brain; thus, these fibers are responsible for a wide range of basic information processing in the brain (Greenstein and Greenstein, 2000). The posterior corona radiata includes axons from and to the cerebral cortex, parietal lobe, and sensorimotor cortex (Dougherty et al., 2007). The dorsal part of the splenium of the corpus callosum connects the bilateral inferior parietal lobule (Pandya et al., 1971). These parietal areas are involved in a wide range of higher-order information processing, such as attention and spatial information processing (Yantis et al., 2002). The positive associations between parents’ average highest educational qualifications and FA in these areas may underlie many of the associations observed between cognitive function and parents’ average highest educational qualifications. However, *post hoc* exploratory analyses showed no associations between the mean DTI values of significant clusters of the educational qualification of parents and psychological correlates of the educational qualification of parents in the expected direction. Therefore, other neural mechanisms that could not be detected with the methods used in the present study may underlie the significant associations between psychological variables and the parents’ educational qualifications. A previous study (Ursache and Noble, 2016b) has shown that parents’ educational qualification positively correlates with similar white matter tracts in the parietal cortex in the left hemisphere and that family income positively correlates with higher FA in areas of the right parahippocampal cingulum and right white matter in the frontal cortex. The present study failed to find significant associations with FA. The reasons for discrepancies between these two studies are unclear. One possible reason could be the lack of statistical power (as well as weak true effects of family SES on FA) to reveal the whole picture of the associations between family SES and FA. Consistent with this speculation, our present data show weak associations between FA and parents’ educational qualification in the frontal cortex and parahippocampal areas.

It is not possible to ascertain the causal mechanism underlying the positive associations among rGMV, FA, and family SES from the cross-sectional design of this study. One possible mechanism is use-dependent neural plasticity. It is known that higher family SES leads to a rich cognitive environment that includes more cognitive stimulation and a better educational environment (Ursache and Noble, 2016a). On the other hand, cognitive stimulation leads to an increase in rGMV and FA, perhaps through increased myelination and tissue components, such as synapses (Takeuchi et al., 2010a). However, other environmental influences, such as nutrition and stress, which are associated with family SES, as well as genetic influences cannot be excluded (Ursache and Noble, 2016a).

The interaction effects of MD, FA, and parents’ average highest educational qualifications were mainly found in the areas of (a) the body of the corpus callosum (Barbas and Pandya, 1984), which connects the bilateral dorsolateral prefrontal cortices that play a key role in the central executive system, and (b) the anterior part of the corona radiata and superior longitudinal fasciculus, which exists between the bilateral lateral prefrontal cortex and anterior cingulate cortex, as well as the parietal area (Pandya et al., 1971); all these areas play a key role in higher-order cognitive functions (Takeuchi et al., 2010a). Similarly, interaction effects between MD and family income were found in the thalamus, which plays a fundamental role in information processing involving the cortex (Sato et al., 2015). The thalamus also plays a fundamental role in coordinating information flow in the brain, integrating broad cognitive processes, such as incoming sensory impulses of pain, and regulating arousal and sleep (for summary, see Sato et al., 2015). The present interaction effects suggest that an association of family SES with higher-order information processing and basic information processing. The interaction effects were mediated by tendencies of negative/positive correlations in males/females for FA and positive/negative correlations in males/females for MD. An increase in FA and decrease in MD may be mediated by increases in myelination and synapses (in the Introduction, see the list of possible tissue mechanisms that impact these values) while the MD changes without FA can be caused by a wide range of neural tissue changes.

We can speculate on why these interaction effects on FA and MD were observed. First, previous animal studies have shown that in response to stressors, females and males show opposite neural changes in some measures. For example, Shors et al. (2001) have reported that in response to stress, females show decreased synapse density, whereas males show increased synapse density. Notably, family SES is associated with stress levels (Ursache and Noble, 2016a). Thus, family SES may impact FA and MD differently in males and females. Second, there may be a sex difference in how family SES is associated with behaviors or traits, which leads to use-dependent plasticity and changes in FA and MD. As shown in the present study, family income was negatively associated with sex role stereotypes in women (which includes the idea that women should not have jobs) but not in men. The egalitarian view regarding sex roles may lead to more eager learning, which may facilitate neural plasticity (Draganski et al., 2006). In addition, parents’ educational qualification was positively associated with more “cognitive” thinking styles or traits (systemizing, critical thinking disposition, and need for cognition). However, there were no significant interaction effects between sex and parents’ educational qualification on these measures after correction for multiple comparisons. Uncorrected, there was a significant interaction effect on systemizing and a trend toward an interaction effect on critical thinking disposition (females partly showed a stronger positive correlation). Traits involving more cognitive activities may lead to an increase in use-dependent plasticity in females. Third, there may be non-linear effects of stress. Stress is assumed to have an inverted-U effect on neural mechanisms, where small amounts of stress cause adaptive changes in neural systems but too much stress is detrimental. On the other hand, higher family SES is associated with lesser stress (Salehi et al., 2010), and human studies have shown that females tend to have more stress events (Hankin et al., 2007). Therefore, greater family SES may reduce males’ stress too much (to too small level) and may not be adaptive in some aspects while for in females things are the opposite and greater family SES may reduce females’ stress to appropriate level. However, our macro-level cross-sectional neuroimaging study cannot determine which of these factors contribute to our findings on the effects of interaction between sex and family SES on FA and MD.

The effects of family SES on brain structure have often been investigated using objective SES methods i.e., family income, income to needs, parents’ education levels; (for review, see Hurt and Betancourt, 2015). On the other hand, for several topics such as health-related outcomes, the subjective effects of SES (i.e., the individual’s perception of his own position in the social hierarchy) are well investigated. Previous studies have shown that the subjective SES are more strongly or independently associated with health-related outcomes (Singh-Manoux et al., 2005; Cohen et al., 2008). Therefore, in some brain structures, especially those related to stress, subjective SES may be more or independently associated with brain imaging measures and the difference between the effects of subjective and objective SES measures on brain imaging measures constitutes an interesting topic for further studies.

The present study had at least one other limitation, i.e., limited sampling. Subjects of this study were young and healthy, consisting of mostly undergraduate and postgraduate students, meaning that they are well educated and the variance of relevant psychological measures may be relatively smaller than that in the general population. This is a common hazard of studies using college students (Jung et al., 2010). This may explain the lack of significant effects of SES measures on some basic cognitive function measures, such as non-verbal reasoning and working memory. In addition, although the present study was focused on the association between family SES and brain structures among imaging measures, when functional activities during working memory and simple cognitive processes [i.e., activities during the 2-back working memory task and the 0-back working memory task (Takeuchi et al., 2018)] are used as dependent variables, no significant main effects of the family SES or of the interaction between family SES and sex were found on such brain activity measures (briefly, the analyses were performed according to our previous study on genotype (Takeuchi et al., 2018), except that the independent variable of interest was replaced from the genotype to either family income or parents’ educational qualifications). The associations between SES measures and psychological/imaging measures may be different in other demographic groups. However, these observations support the importance of assessing the effects of SES measures on a wide range of measures related to traits, states, and effects and relevant neural mechanisms. Also, effects of environment on cognitive mechanisms may be more evident during childhood and in disadvantaged environments and this fact might lead to smaller effects in the present study involving educated samples in developed countries (Haworth et al., 2010). On the other hand, our sample involved young adults and the development of the brain is still continuing and sex differences of developmental timing exist and any sex differences observed in this study may have something to do with sex differences of developmental timing (Taki et al., 2013). Nonetheless, future studies are needed to elucidate the effects of SES measures on the psychological/imaging measures in the general population. Also, as were the cases of all studies in the field, we used SES measures, and any associations between SES measures and outcome measures can be due to intermediate factors. These intermediate factors of course include genetic factors. Since it is a common matter in studies of correlates of SES measures, we did not even try to control these intermediate factors, and future studies can reveal them.

## Conclusion

In conclusion, previous studies have revealed that family SES is positively associated with greater cognitive abilities, brain volume, and FA (white matter structural property). These findings were at least partly replicated in the present study involving young adults with a high educational qualification. The present study revealed that family income was associated with microstructural properties of the thalamus (females with greater family income show lower MD and males showed opposite patterns), which plays a key role in basic information processing in the brain. Furthermore, parents’ educational qualification was associated with sex-specific microstructural properties of white matter areas that play a key role in higher-order cognitive activities (females with family education level show greater FA or lower MD in females and opposite in males). These results suggest that sex-specific neural and/or cognitive mechanisms are associated with family SES and neural tissue characteristics. Family income also showed sex-specific associations with sex role stereotypes (females with more family income showed reduced sex role stereotypes). In addition, family SES was associated with a wide range of psychological properties related to well-being. Family income was also associated with greater nationalism and competitive achievement motivation, and parents’ educational qualification was associated with traits related to more cognitive thinking styles. These results suggest that family SES impacts a wide range of traits.

## Informed Consent

Informed consent was obtained from all individual participants included in the study.

## Ethics Statement

All procedures performed in studies involving human participants were in accordance with the ethical standards of the institutional and/or national research committee and with the 1964 Helsinki declaration and its later amendments or comparable ethical standards.

## Author Contributions

HT, YT, and RK designed the study. HT, AS, RN, YK, SN, CM, KI, RY, YY, SH, TA, YS, KS, TN, SI, SY, and DM collected the data. HT analyzed the data and prepared the manuscript.

## Conflict of Interest Statement

The authors declare that the research was conducted in the absence of any commercial or financial relationships that could be construed as a potential conflict of interest.

## Footnotes

1 http://dbm.neuro.uni-jena.de/tfce/
