# Supplementary material for: The Effects of Family Socioeconomic Status on Psychological and Neural Mechanisms as Well as Their Sex Differences
Source: Front Hum Neurosci. 2019 Jan 18;12:543. doi: 10.3389/fnhum.2018.00543 (PMC6345688; doi:10.3389/fnhum.2018.00543)
Supplement: Supplementary file 1 [file Data_Sheet_1.docx]

**Supplemental online material**

**Supplemental Methods**

**Subjects.** The present study, which is a part of an ongoing project to investigate the association between brain imaging, cognitive function, and aging, included relevant SES measures and imaging data from 1216 healthy, right-handed individuals (702 men and 514 women). This project has started in 2008 and still is continuing. The mean age of the subjects was 20.7 years [standard deviation (SD), 1.8; age range: 18-27 years old]. The following descriptions were mostly reproduced from another study of ours from the same project using the exactly same methods regarding these issues ([Takeuchi et al., 2015a](#_ENREF_49)). Some of the subjects who took part in this study also became subjects of our intervention studies (psychological data and imaging data recorded before the intervention were used in this study)([Takeuchi et al., 2014](#_ENREF_48)). Psychological tests and MRI scans not described in this study were performed together with those described in this study. All subjects were university students, postgraduates, or university graduates of less than one year’s standing. Four subjects had graduated at the time of the experiment and their removal did not alter the significance of the present findings. All subjects had normal vision and none had a history of neurological or psychiatric illness. Handedness was evaluated using the Edinburgh Handedness Inventory ([Oldfield, 1971](#_ENREF_25)). Written informed consent was obtained from each subject. For nonadult subjects, written informed consent was obtained from their parents (guardians). This study was approved by the Ethics Committee of Tohoku University.

Subjects were instructed to get sufficient sleep, maintain their conditions, eat sufficient breakfast, and to consume their normal amounts of caffeinated foods and drinks in the day of cognitive tests and MRI scans. In addition, subjects were instructed to avoid alcohol the night before the assessment.

Psychological measures.

The following neuropsychological tests and questionnaires were administered to subjects:

(A) Raven’s Advanced Progressive Matrices (RAPM) ([Raven, 1998](#_ENREF_26)), a non-verbal reasoning task; (B) Tanaka B-type intelligence test (TBIT) type 3B ([Tanaka et al., 2003](#_ENREF_57)). This test is for examinees in their 3rd-year of junior high school and older. This test is a nonverbal mass intelligence test which does not include story problems but uses figures, single numbers, and letters as stimuli. In all subtests, subjects had to complete as many problems as possible within a certain time (a few minutes); (C) A reading comprehension task ([Kondo et al., 2003](#_ENREF_17)). This test involves several articles. Each article has four questions and each question has five choices of answers. Subjects have 13 minutes to read the articles and answer the questions; (D) the S-A creativity test ([Society_For_Creative_Minds, 1969](#_ENREF_35)), a measure of creativity measured by divergent thinking (CMDT). A detailed discussion of the psychometric properties of this instrument and how it was developed is found in the technical manual of this test ([Society_For_Creative_Minds, 1969](#_ENREF_35)). The test was used to evaluate creativity through divergent thinking ([Society_For_Creative_Minds, 1969](#_ENREF_35)). Subjects were asked to generate as many answers as possible to specific questions. For more details including the psychometric properties of this test, sample answers to the questionnaire, and the manner in which they were scored, see our previous works ([Takeuchi et al., 2010a](#_ENREF_39);[c](#_ENREF_41)); [E] A (computerized) digit span task, a verbal WM task ([for the detail of this task, seeTakeuchi et al., 2011b](#_ENREF_43)). [F] SQ and EQ questionnaires. Japanese versions ([Wakabayashi et al., 2007](#_ENREF_61)) of the SQ and EQ questionnaires ([Baron-Cohen et al., 2003](#_ENREF_4);[Baron-Cohen and Wheelwright, 2004](#_ENREF_5)) were administered. The EQ score was used as an index of empathizing (drive to identify the mental status of other individuals), and the SQ score was used as an index of systemizing (drive to analyze a system); [G] the Japanese version of the Emotional Intelligence Scale ([Fukunishi et al., 2001](#_ENREF_10);[Uchiyama et al., 2001](#_ENREF_59)), a measure of emotional intelligence. The Emotional Intelligence Scale is a self-reported measurement that provides an estimate of emotional and social intelligence. Total score of this scale was used in this study; (G) General Health Questionnaire 30 (GHQ30), a measure of mental health ([Nakagawa and Daibo, 1996](#_ENREF_22)); (H) WHOQOL-26 ([Tazaki and Nakane, 2007](#_ENREF_58)), the Japanese version of the QOL Scale; (I) the Scale for Critical Thinking Disposition ([Hirayama and Kusumi, 2004](#_ENREF_13)), a measure of disposition toward critical thinking; (J) the cognitive reflectivity–impulsiveness questionnaire ([Takigiku and Sakamoto, 1991](#_ENREF_56)), a measure of individual differences in reflectivity and impulsivity; (K) the Japanese version of the Need for Cognition Scale ([Kouyama and Fujiwara, 1991](#_ENREF_18)), a measure of the tendency of an individual to engage in and enjoy thinking; (L) the Japanese version of the Achievement Motivation Scale ([Horino and Mori, 1991](#_ENREF_14)), a measure of individual achievement motivation. This questionnaire evaluates two psychometrically derived achievement motivations, i.e., self-fulfillment achievement motivation (SFAM) and competitive achievement motivation (CAM) ([Horino and Mori, 1991](#_ENREF_14)). SFAM is achievement motivation directed at pursuing goals evaluated by one’s own standards of achievement regardless of the values of others and the society. CAM is achievement motivation directed at seeking social prestige by defeating others and achieving better results than others; (M) the Japanese version ([Yamamoto, 1982](#_ENREF_62)) of the Rosenberg Self-Esteem Scale (RSES) ([Rosenberg, 1965](#_ENREF_27)), a measure of global trait self-esteem; (N) the National Identity Scale ([Karasawa, 2002](#_ENREF_16)), a measure of individual nationalism and patriotism. This questionnaire is a self-reported measure of Japanese national identity and has been used for measuring Japanese national identity; (O) the Japanese version of the Optimism Scale ([Nakamura, 2000](#_ENREF_23)), a questionnaire to measure individual optimism and pessimism; (P) the Japanese version ([Narita et al., 1995](#_ENREF_24)) of the General Self-Efficacy Scale ([Sherer et al., 1982](#_ENREF_31)), a questionnaire to measure individual general self-efficacy. General self-efficacy is defined as individuals’ perception of their ability to perform across various different situations ([Judge et al., 1998](#_ENREF_15)); (Q) the Japanese version ([Kudoh and Nishikawa, 1983](#_ENREF_19)) of the third version of the UCLA Loneliness Scale ([Russell et al., 1980](#_ENREF_28)), a questionnaire to measure social isolation and loneliness; (R) the Japanese version ([Aratake et al., 2007](#_ENREF_1)) of the checklist individual strength questionnaire (CIS), developed by Vercoule et al. ([1994](#_ENREF_60)), a measure of chronic fatigue; (S) the Japanese version ([Hayashi and Takimoto, 1991](#_ENREF_12)) of the Beck Depression Inventory, a questionnaire that measures depression states ([Beck et al., 1979](#_ENREF_6)); (T) the Japanese ([Shimizu and Imae, 1981](#_ENREF_32)) version of State-Trait Anxiety Inventory (STAI) ([Spielberger et al., 1999](#_ENREF_36)), a questionnaire that measures state and trait anxiety; (U) the shortened Japanese version ([Yokoyama, 2005](#_ENREF_63)) of the Profile of Mood States (POMS) questionnaire ([McNair et al., 1992](#_ENREF_21)), a measure of each participant’s mood during the preceding week. The total score (total mood disturbance) was used in this study; (V) The Japanese version of the NEO Five-Factor Inventory (NEO-FFI) ([Costa and McCrae, 1992](#_ENREF_9)), a measure of five basic personalities, including neuroticism, extraversion, openness, agreeableness, and conscientiousness; (W) the Japanese version ([Sawada and Arai, 2002](#_ENREF_30)) of the Dispositional Envy Scale ([Smith et al., 1999](#_ENREF_33)), a questionnaire to measure the propensity for envy; (X) the Preoccupation Scale ([Sakamoto, 1998](#_ENREF_29)), which includes the External-Preoccupation Scale that measures the maintenance of external focus on a specific object, and the Self-Preoccupation Scale, which measures both the degree and duration of self-focusing; (Y) the Scale of Egalitarian Sex Role Attitudes-Short Form (SESRA-S) ([Suzuki, 1994](#_ENREF_37)), a self-report questionnaire used to measure an individual’s sex-role egalitarianism (SRE), which is the belief that the sex of an individual should not influence the perception of his/her rights, abilities, obligations, and opportunities ([Ben-David and Schneider, 2005](#_ENREF_7)); and (Z) Scale of life events in interpersonal and achievement domains ([Takahira, 1998](#_ENREF_38)), a measure of students’ negative and positive events. The scores were divided into negative life event scores and positive life event scores.

**The rationale and basic explanation of the FDR test.** FDR is the error rate in the set of comparisons that are called signiﬁcant, or, in other words, the proportion of comparisons which are wrongly called signiﬁcant In other words, among the multiple tested results, 5% of the results determined to be significant through this method, are not truly significant. In FDR testing, if there is truly no signal (nonnoise) anywhere in the tested results, an FDR-controlling method has the same control as a family-wise error correction. FDR-based methods have been shown to be more powerful and sensitive than other available approaches to multiple statistical testing (See [Benjamini and Hochberg, 1995 for a full discussion](#_ENREF_8);[Genovese et al., 2002](#_ENREF_11)). The description is reproduced from our previous study ([Takeuchi et al., 2013c](#_ENREF_46)).

**Details of diffusion image acquisition.** There are acquisitions for phase correction and for signal stabilization and these are not used as reconstructed images. MD and FA maps were calculated from the collected images using a commercially available diffusion tensor analysis package on the MR consol. This practice has been used in many of our previous studies ([Takeuchi et al., 2010b](#_ENREF_40);[Takeuchi et al., 2010c](#_ENREF_41); [2011a](#_ENREF_42);[Takeuchi et al., 2013b](#_ENREF_45);[Takeuchi et al., 2013d](#_ENREF_47)). Furthermore, the results of analyses using these image-generated results were congruent with those of previous studies in which other methods were used ([Barnea-Goraly et al., 2005](#_ENREF_3);[Taki et al., 2013](#_ENREF_55)), suggesting the validity of this method. These procedures involved correction for motion and distortion caused by eddy currents. Calculations were performed according to a previously proposed method ([Le Bihan et al., 2001](#_ENREF_20)). The descriptions in this subsection were largely reproduced from our previous study that used the same methods ([Takeuchi et al., 2016](#_ENREF_52)).

**Pre-processing of structural data.** The methods for the preprocessing of T1WIs were described in our previous study and reproduced below ([Takeuchi et al., 2018](#_ENREF_53)). Preprocessing of the structural data was performed using Statistical Parametric Mapping software (SPM12; Wellcome Department of Cognitive Neurology, London, UK) implemented in Matlab (Mathworks Inc., Natick, MA, USA). Using the new segmentation algorithm implemented in SPM12, T1-weighted structural images of each individual were segmented into 6 tissues. In this new segmentation process, default parameters were used, except that the Thorough Clean option was used to eliminate any odd voxel, affine regularization was performed with the International Consortium for Brain Mapping template for East Asian brains, and the sampling distance was set at 1 mm. We then proceeded to the diffeomorphic anatomical registration through exponentiated lie algebra (DARTEL) registration process implemented in SPM12. We used DARTEL import images of the 2 TPMs from the abovementioned new segmentation process. First, the template for the DARTEL procedures was created using imaging data from 800 participants (400 males and 400 females). The following methods were the same as in our previous study and descriptions were reproduced from our previous study ([Takeuchi et al., 2015c](#_ENREF_51)). Next, using this existing template, the DARTEL procedures were performed for all of the subjects in the present study. In these procedures, default parameter settings were used. The resulting images were spatially normalized to the Montreal Neurological Institute (MNI) space to give images with 1.5 × 1.5 × 1.5 mm^3^ voxels. In addition, we performed a volume change correction (modulation) by modulating each voxel with the Jacobian determinants derived from spatial normalization, which allowed us to determine regional differences in the absolute amount of brain tissue ([Ashburner and Friston, 2000](#_ENREF_2)). Subsequently, normalized rGMV, rWMV, and rCSF volume images were smoothed by convolving them with an isotropic Gaussian kernel of 8 mm full width at half maximum (FWHM).

**Pre-processing of diffusion data**

The methods for the preprocessing of diffusion data were described in our previous study and reproduced below ([Takeuchi et al., in press](#_ENREF_54)). Preprocessing and analysis of diffusion data were performed using Statistical Parametric Mapping (SPM) 8 implemented in Matlab. Using a previously validated two-step new segmentation algorithm of diffusion images and the previously validated diffeomorphic anatomical registration through exponentiated lie algebra (DARTEL)-based registration process ([Takeuchi et al., 2013b](#_ENREF_45)), all images, including gray matter segment [regional gray matter density (rGMD) map], white matter segment [regional white matter density (rWMD) map], cerebrospinal fluid (CSF) segments [regional CSF density (rCSFD) map] of diffusion images, were normalized. The voxel size of normalized FA images and MD images and segmented images, was 1.5 × 1.5 × 1.5 mm^3^. See our previous work ([Takeuchi et al., 2013a](#_ENREF_44)) for the details of these procedures including the information of the template.

Next, we created average images of normalized rGMD and rWMD images from the normalized rGMD and rWMD images from the subset of the entire sample (63 subjects) ([Takeuchi et al., 2013b](#_ENREF_45)). Subsequently, for the analyses of MD images from the normalized images of the (a) MD, (b) rGMD, and (c) rCSFD maps, we created images where areas that were not strongly likely to be gray or white matter in our averaged normalized rGMD and rWMD images (defined by “gray matter tissue probability + white matter tissue probability < 0.99”) were removed (to exclude the strong effects of CSF on MD throughout analyses). These images were then smoothed (8 mm full-width half-maximum) and carried through to the second-level analyses of MD.

Next, from the average image of normalized WM segmentation images from the 63 subjects mentioned above and from the created mask image consisting of voxels with a WM signal intensity > 0.99. The thresholding procedures of the MD and FA images were performed with these procedures with the template after careful normalization of each individual’s images and not with the individual images’ signal intensities. We then applied this mask image to the normalized FA image; therefore, we retained only areas that are highly likely to be white matter from the normalized FA images. These images were smoothed (6 mm full-width half-maximum) and carried through to the second-level analyses of FA.

A smaller smoothing value was chosen in FA because although 8 mm FWHM is relatively common in imaging analyses, FA values differ substantially among adjacent tracts, and use of larger smoothing variables contaminate signals from different tracts more and this makes larger smoothing values unfavorable.

These preprocessing procedures utilized the new SPM8 segmentation and modified DARTEL procedures that take into account the FA signal distribution in white matter tissues and that effectively solve the misalignment problem of voxel-based analyses of FA ([Smith et al., 2006](#_ENREF_34)). By applying the masks of tissue probability >0.99 before smoothing, this method also effectively alleviates the partial volume effects and the problems of signal contamination from other tissues in voxel-based analyses of FA ([Smith et al., 2006](#_ENREF_34)). For the quantitative and visual demonstration of the validity of these methods, see supplemental online material for our previous work ([Takeuchi et al., 2013a](#_ENREF_44)).

We did not use T1 weighted structural images for these preprocessing procedures. As described previously “*This is because T1 weighted structural images and EPI images have apparent differences due to the distortion caused by 3T MRI and simply it is apparently not suited for the accurate and precise segmentation and normalization images of MD maps.*” ([Takeuchi et al., 2015b](#_ENREF_50)).

**The reasons why we were using different versions of SPM.**

We used SPM12 for VBM preprocessing and SPM8 for statistical analyses because of the compatibility of the software that we used for permutation-based statistics (TFCE) and of the script we used for the statistical analyses. If permutation tests were used, the results were not affected by the version of SPM. Also, in the case of preprocessing of DTI images, apparently, our preprocessing procedures did not go well with SPM12 and quality of the resultant images was not good.

**References**

Aratake, Y., Tanaka, K., Wada, K., Watanabe, M., Katoh, N., Sakata, Y., and Aizawa, Y. (2007). Development of Japanese version of the checklist individual strength questionnaire in a working population. *J Occup Health* 49**,** 453-460.

Ashburner, J., and Friston, K.J. (2000). Voxel-based morphometry-the methods. *Neuroimage* 11**,** 805-821.

Barnea-Goraly, N., Menon, V., Eckert, M., Tamm, L., Bammer, R., Karchemskiy, A., Dant, C.C., and Reiss, A.L. (2005). White matter development during childhood and adolescence: a cross-sectional diffusion tensor imaging study. *Cerebral cortex* 15**,** 1848-1854.

Baron-Cohen, S., Richler, J., Bisarya, D., Gurunathan, N., and Wheelwright, S. (2003). The systemizing quotient: an investigation of adults with Asperger syndrome or high-functioning autism, and normal sex differences. *Philosophical Transactions of the Royal Society of London. Series B: Biological Sciences* 358**,** 361-374.

Baron-Cohen, S., and Wheelwright, S. (2004). The empathy quotient: an investigation of adults with Asperger syndrome or high functioning autism, and normal sex differences. *Journal of Autism and Developmental Disorders* 34**,** 163-175.

Beck, A.T., Rush, A.J., Shaw, B.F., and Emery, G. (1979). *Cognitive therapy of depression.* Guilford press.

Ben-David, S., and Schneider, O. (2005). Rape perceptions, gender role attitudes, and victim-perpetrator acquaintance. *Sex Roles* 53**,** 385-399.

Benjamini, Y., and Hochberg, Y. (1995). Controlling the false discovery rate: a practical and powerful approach to multiple testing. *Journal of the Royal Statistical Society. Series B (Methodological)* 57**,** 289-300.

Costa, P.T., and Mccrae, R.R. (1992). *Professional manual: revised NEO personality inventory (NEO-PI-R) and NEO five-factor inventory (NEO-FFI).* Odessa, FL: Psychological Assessment Resources.

Fukunishi, I., Wise, T.N., Sheridan, M., Shimai, S., Otake, K., Utsuki, N., and Uchiyama, K. (2001). Validity and reliability of the Japanese version of the Emotional Intelligence Scale among college students and psychiatric outpatients. *Psychological reports* 89**,** 625-632.

Genovese, C.R., Lazar, N.A., and Nichols, T. (2002). Thresholding of statistical maps in functional neuroimaging using the false discovery rate. *Neuroimage* 15**,** 870-878.

Hayashi, K., and Takimoto, T. (1991). An Examination of Beck Depression Inventory (1978) and an Analysis of Relation between Depressive Tendencies and Grade of Self-efficacy (Cultural and Social Science). *Memoirs of Shiraume Gakuen College* 27**,** 43-52.

Hirayama, R., and Kusumi, T. (2004). Effect of Critical Thinking Disposition on Interpretation of Controversial Issues, Evaluating Evidences and Drawing Conclusions. *The Japanese Journal of Educational Psychology* 52**,** 186-198.

Horino, M., and Mori, K. (1991). The effects of achievement motivation on relationships between depression and social support. *Kyoikushinrigakukenkyu (Educational Psychology Research)* 39**,** 308-315.

Judge, T.A., Erez, A., and Bono, J.E. (1998). The Power of Being Positive: The Relation Between Positive Self-Concept and job Performance. *Human Performance* 11**,** 167-187.

Karasawa, M. (2002). Patriotism, nationalism, and internationalism among Japanese citizens: An etic–emic approach. *Political Psychology* 23**,** 645-666.

Kondo, H., Morishita, M., Ashida, K., and Osaka, N. (2003). Reading Comprehension and Working Memory--Structural Equation Modeling Approach. *The Japanese Journal of Psychology* 73**,** 480-487.

Kouyama, T., and Fujiwara, T. (1991). A basic study of the Need for Cognition Scale [in Japanese]. *Japanese Journal of Social Psychology* 6**,** 184-192.

Kudoh, T., and Nishikawa, M. (1983). A study of the feeling of loneliness: I. The reliability and validity of the revised UCLA Loneliness Scale. *Japanese Journal of Experimental Social Psychology*.

Le Bihan, D., Mangin, J.F., Poupon, C., Clark, C.A., Pappata, S., Molko, N., and Chabriat, H. (2001). Diffusion tensor imaging: concepts and applications. *Journal of Magnetic Resonance Imaging* 13**,** 534-546.

Mcnair, D.M., Lorr, M., and Droppleman, L.F. (1992). *Profile of mood states.* San Diego, California: Educational and Industrial Testing Service.

Nakagawa, Y., and Daibo, I. (1996). *Japanese version GHQ30 (in Japanese).* Tokyo: Nihon Bunka Kagakusya.

Nakamura, Y. (2000). *Psychological individual differences in the interpersonal situation (in Japanese).* Brain Syuppan.

Narita, K., Shimonaka, Y., Nakazato, K., Kawaai, C., Sato, S., and Osada, Y. (1995). A Japanese version of the generalized self-efficacy scale - Scale utility from the life-span perspective. *Japanese Journal of Educational Psychology* 43**,** 306-314.

Oldfield, R.C. (1971). The assessment and analysis of handedness: the Edinburgh inventory. *Neuropsychologia* 9**,** 97-113.

Raven, J. (1998). *Manual for Raven's progressive matrices and vocabulary scales.* Oxford: Oxford Psychologists Press.

Rosenberg, M. (1965). *Society and the adolescent self-image.* Princeton, NJ Princeton University Press. .

Russell, D., Peplau, L.A., and Cutrona, C.E. (1980). The revised UCLA Loneliness Scale: concurrent and discriminant validity evidence. *J Pers Soc Psychol* 39**,** 472-480.

Sakamoto, S. (1998). The Preoccupation Scale: Its development and relationship with depression scales. *Journal of Clinical Psychology* 54**,** 645-654.

Sawada, M., and Arai, K. (2002). Dispositional Envy, Domain Importance, and Obtainability of Desired Objects: Selection of Strategies for Coping with Envy (in Japanese). *The Japanese Journal of Educational Psychology* 50**,** 246-256.

Sherer, M., Maddux, J.E., Mercandante, B., Prentice-Dunn, S., Jacobs, B., and Rogers, R.W. (1982). The self-efficacy scale: Construction and validation. *Psychological reports* 51**,** 663-671.

Shimizu, H., and Imae, K. (1981). Development of Japanese version of STATE-TRAIT ANXIETY INVENTORY for university. *The Japanese journal of educational psychology* 29**,** 348-353.

Smith, R.H., Parrott, W.G., Diener, E.F., Hoyle, R.H., and Kim, S.H. (1999). Dispositional envy. *Personality and Social Psychology Bulletin* 25**,** 1007-1020.

Smith, S.M., Jenkinson, M., Johansen-Berg, H., Rueckert, D., Nichols, T.E., Mackay, C.E., Watkins, K.E., Ciccarelli, O., Cader, M.Z., and Matthews, P.M. (2006). Tract-based spatial statistics: voxelwise analysis of multi-subject diffusion data. *Neuroimage* 31**,** 1487-1505.

Society_for_Creative_Minds (1969). *Manual of S-A creativity test.* Tokyo, Japan: Tokyo shinri Corporation.

Spielberger, C.D., Sydeman, S.J., Owen, A.E., and Marsh, B.J. (1999). "Measuring anxiety and anger with the State-Trait Anxiety Inventory (STAI) and the State-Trait Anger Expression Inventory (STAXI)," in *The use of psychological testing for treatment planning and outcomes assessment (2nd ed.),* ed. M. Maruish. (Mahwah, NJ, US: Lawrence Erlbaum Associates Publishers), 993-1021.

Suzuki, A. (1994). Construction of a short-form of the scale of egalitarian sex role attitudes (SESRA-S)]. *Shinrigaku kenkyu: The Japanese journal of psychology* 65**,** 34-41.

Takahira, M. (1998). Construction of a scale of life events in interpersonal and achievement domains for undergraduate students [in Japanese]. *Japanese Journal of Social Psychology* 14**,** 12-24.

Takeuchi, H., Taki, Y., Sassa, Y., Hashizume, H., Sekiguchi, A., Fukushima, A., and Kawashima, R. (2010a). Regional gray matter volume of dopaminergic system associate with creativity: Evidence from voxel-based morphometry *Neuroimage* 51**,** 578-585.

Takeuchi, H., Sekiguchi, A., Taki, Y., Yokoyama, S., Yomogida, Y., Komuro, N., Yamanouchi, T., Suzuki, S., and Kawashima, R. (2010b). Training of Working Memory Impacts Structural Connectivity. *Journal of Neuroscience* 30**,** 3297-3303.

Takeuchi, H., Taki, Y., Sassa, Y., Hashizume, H., Sekiguchi, A., Fukushima, A., and Kawashima, R. (2010c). White matter structures associated with creativity: Evidence from diffusion tensor imaging. *Neuroimage* 51**,** 11-18.

Takeuchi, H., Taki, Y., Sassa, Y., Hashizume, H., Sekiguchi, A., Fukushima, A., and Kawashima, R. (2011a). Verbal working memory performance correlates with regional white matter structures in the fronto-parietal regions. *Neuropsychologia* 49**,** 3466-3473

Takeuchi, H., Taki, Y., Hashizume, H., Sassa, Y., Nagase, T., Nouchi, R., and Kawashima, R. (2011b). Failing to deactivate: the association between brain activity during a working memory task and creativity. *Neuroimage* 55**,** 681-687.

Takeuchi, H., Taki, Y., Sassa, Y., Hashizume, H., Sekiguchi, A., Nagase, T., Nouchi, R., Fukushima, A., and Kawashima, R. (2013a). White matter structures associated with empathizing and systemizing in young adults. *Neuroimage* 77**,** 222-236.

Takeuchi, H., Taki, Y., Thyreau, B., Sassa, Y., Hashizume, H., Sekiguchi, A., Nagase, T., Nouchi, R., Fukushima, A., and Kawashima, R. (2013b). White matter structures associated with empathizing and systemizing in young adults. *Neuroimage* 77**,** 222-236.

Takeuchi, H., Taki, Y., Sekiguchi, A., Nouchi, R., Kotozaki, Y., Nakagawa, S., Miyauchi, C.M., Iizuka, K., Yokoyama, R., Shinada, T., Yamamoto, Y., Hanawa, S., Araki, T., Hashizume, H., Sassa, Y., and Kawashima, R. (2013c). Association of hair iron levels with creativity and psychological variables related to creativity. *Frontiers in Human Neuroscience* 7, Article 875**,** 1-9.

Takeuchi, H., Taki, Y., Sassa, Y., Hashizume, H., Sekiguchi, A., Nagase, T., Nouchi, R., Fukushima, A., and Kawashima, R. (2013d). White matter structures associated with emotional intelligence: Evidence from diffusion tensor imaging. *Human brain mapping* 34**,** 1025-1034.

Takeuchi, H., Taki, Y., Nouchi, R., Hashizume, H., Sekiguchi, A., Kotozaki, Y., Nakagawa, S., Miyauchi, C.M., Sassa, Y., and Kawashima, R. (2014). Effects of Multitasking-Training on Gray Matter Structure and Resting State Neural Mechanisms. *Human Brain Mapping* 35**,** 3646-3660.

Takeuchi, H., Taki, Y., Nouchi, R., Sekiguchi, A., Hashizume, H., Sassa, Y., Kotozaki, Y., Miyauchi, C.M., Yokoyama, R., Iizuka, K., Seishu, N., Tomomi, N., Kunitoki, K., and Kawashima, R. (2015a). Degree centrality and fractional amplitude of low-frequency oscillations associated with Stroop interference. *Neuroimage* 119**,** 197-209.

Takeuchi, H., Taki, Y., Sekuguchi, A., Hashizume, H., Nouchi, R., Sassa, Y., Kotozaki, Y., Miyauchi, C.M., Yokoyama, R., Iizuka, K., Nakagawa, S., Nagase, T., Kunitoki, K., and Kawashima, R. (2015b). Mean diffusivity of globus pallidus associated with verbal creativity measured by divergent thinking and creativity-related temperaments in young healthy adults. *Human Brain Mapping* 36**,** 1808-1827.

Takeuchi, H., Taki, Y., Nouchi, R., Hashizume, H., Sekiguchi, A., Kotozaki, Y., Nakagawa, S., Miyauchi, C.M., Sassa, Y., and Kawashima, R. (2015c). The structure of the amygdala associates with human sexual permissiveness: Evidence from voxel-based morphometry. *Human Brain Mapping* 36**,** 440-448.

Takeuchi, H., Taki, Y., Hashizume, H., Asano, K., Asano, M., Sassa, Y., Yokota, S., Kotozaki, Y., Nouchi, R., and Kawashima, R. (2016). Impact of videogame play on the brain’s microstructural properties: Cross-sectional and longitudinal analyses. *Molecular Psychiatry* 21**,** 1781-1789.

Takeuchi, H., Taki, Y., Nouchi, R., Yokoyama, R., Kotozaki, Y., Nakagawa, S., Sekiguchi, A., Iizuka, K., Yamamoto, Y., Hanawa, S., Araki, T., Miyauchi, C.M., Shinada, T., Yokota, S., Daniele, M., and Kawashima, R. (2018). Allergic tendencies are associated with larger gray matter volumes. *Scientific Reports* 8**,** Article number: 3694.

Takeuchi, H., Tomita, H., Taki, Y., Kikuchi, Y., Ono, C., Yu, Z., Sekiguchi, A., Nouchi, R., Kotozaki, Y., Nakagawa, S., Miyauchi, C.M., Iizuka, K., Yokoyama, R., Shinada, T., Yamamoto, Y., Hanawa, S., Araki, T., Kunitoki, K., Sassa, Y., and Kawashima, R. (in press). Effects of interaction between the BDNF Val66Met polymorphism and daily physical activity on mean diffusivity *Brain Imaging and Behavior*.

Taki, Y., Thyreau, B., Hashizume, H., Sassa, Y., Takeuchi, H., Wu, K., Kotozaki, Y., Nouchi, R., Asano, M., and Asano, K. (2013). Linear and curvilinear correlations of brain white matter volume, fractional anisotropy, and mean diffusivity with age using voxel-based and region of interest analyses in 246 healthy children. *Human Brain Mapping* 34**,** 1842-1856.

Takigiku, K., and Sakamoto, A. (Year). "Cognitive reflectivity–The making of impulsiveness standard–study about the reliability and appropriateness", in: *The 38th Annual Meeting of the Japanese Group Dynamics Association collected papers*), 39-40.

Tanaka, K., Okamoto, K., and Tanaka, H. (2003). *Manual of New Tanaka B type intelligence test.* Tokyo: Kaneko Syobo.

Tazaki, M., and Nakane, M. (2007). *Revised manual of WHO QOL 26.* Tokyo: Kaneko-syobou.

Uchiyama, K., Shimai, T., Utsuki, N., and Otake, K. (2001). *EQS manual.* Tokyo: Jitsumukyoiku Syuppan (Practical Education Press).

Vercoulen, J.H., Swanink, C.M., Fennis, J.F., Galama, J.M., Van Der Meer, J.W., and Bleijenberg, G. (1994). Dimensional assessment of chronic fatigue syndrome. *J Psychosom Res* 38**,** 383-392.

Wakabayashi, A., Baron-Cohen, S., Uchiyama, T., Yoshida, Y., Kuroda, M., and Wheelwright, S. (2007). Empathizing and systemizing in adults with and without autism spectrum conditions: cross-cultural stability. *Journal of Autism and Developmental Disorders* 37**,** 1823-1832.

Yamamoto, M., Matsui, Y., Yamanari, Y. (1982). Ninchi sareta jiko no shosokumen no kouzou. [structure of self-recognition]. *Japanese Journal of Educational Psychology* 30**,** 64-68.

Yokoyama, K. (2005). *POMS Shortened Version (in Japanese).* Tokyo: Kanekoshobo.
